# Photodynamic studies reveal rapid formation and appreciable turnover of tau inclusions

**DOI:** 10.1007/s00401-021-02264-9

**Published:** 2021-01-26

**Authors:** Cara L. Croft, Marshall S. Goodwin, Daniel H. Ryu, Christian B. Lessard, Giancarlo Tejeda, Marc Marrero, Ava R. Vause, Giavanna Paterno, Pedro E. Cruz, Jada Lewis, Benoit I. Giasson, Todd E. Golde

**Affiliations:** 1 Department of Neuroscience, College of Medicine, University of Florida, Gainesville, FL 32610 USA; 2 Center for Translational Research in Neurodegenerative Disease, College of Medicine, University of Florida, Gainesville, FL 32610 USA; 3 McKnight Brain Institute, College of Medicine, University of Florida, Gainesville, FL 32610 USA; 4 UK Dementia Research Institute at University College London, London, UK; 5 Department of Neurology, College of Medicine, University of Florida, Gainesville, FL 32610 USA

**Keywords:** Microtubule-associated protein tau, Recombinant adeno-associated viruses, Tau inclusion turnover, Brain slice culture, Optical pulse labeling

## Abstract

Accumulation of the tau protein in fibrillar intracellular aggregates is a defining feature of multiple neurodegenerative diseases collectively referred to as tauopathies. Despite intensive study of tau, there is limited information on the formation and clearance dynamics of tau inclusions. Using rAAV vectors to mediate expression of Dendra2-tagged human wild-type, P301L and pro-aggregant P301L/S320F tau proteins, with and without the addition of exogenous tau fibrillar seeds, we evaluated tau inclusion dynamics in organotypic brain slice culture (BSC) models using long-term optical pulse labeling methodology. Our studies reveal that tau inclusions typically form in 12–96 h in tauopathy BSC models. Unexpectedly, we demonstrate appreciable turnover of tau within inclusions with an average half-life of ~ 1 week when inclusions are newly formed. When BSCs with inclusions are aged in culture for extended periods, tau inclusions continue to turnover, but their half-lives increase to ~ 2 weeks and ~ 3 weeks after 1 and 2 months in culture, respectively. Individual tau inclusions can be long-lived structures that can persist for months in these BSC models and for even longer in the human brain. However, our data indicate that tau inclusions, are not ‘tombstones’, but dynamic structures with appreciable turnover. Understanding the cellular processes mediating this inclusion turnover may lead to new therapeutic strategies that could reverse pathological tau inclusion formation.

**Supplementary Information:**

The online version contains supplementary material available at 10.1007/s00401-021-02264-9.

## Introduction

Neurodegenerative tauopathies including Alzheimer’s disease (AD) are tightly associated with the intracellular accumulation of aberrantly aggregated tau protein within the brain [20, 24]. Unbound microtubule-associated protein tau (MAPT) exists physiologically as a soluble unstructured protein, but under pathogenic conditions tau assembles into insoluble β sheet-rich amyloidogenic fibrillar inclusions in AD and other tauopathies [20, 24, 31]. Notably, these inclusions are extensively post-translationally modified, with prominent hyperphosphorylation. In AD, the accumulation of tau in neurofibrillary tangles (NFTs) significantly correlates with cognitive decline, brain atrophy and neuronal loss [2, 26]. Conversely, it is also recognized that tau inclusions may remain stable in neurons for decades in humans [3] and more recent evidence suggests that neuronal function is not compromised in tau transgenic mice with NFT pathology [36, 44, 47]. Genetic, modeling and pathological studies all support the therapeutic targeting of tau in neurodegenerative disorders, despite the fact that we still have a limited understanding of the mechanisms that underlie tau accumulation and tau-associated neurodegeneration. Prion-like mechanisms of seeding and propagation of tau inclusions are now well-established in many model systems, and are proposed as mechanisms for the spread of tau pathology [23]. Indeed, both recombinant and AD-derived tau can, with varying efficiencies, seed tau pathology in both tau over-expressing transgenic models [8, 9, 19, 23, 37, 43] and non-transgenic models [23, 30, 37, 43, 50]. To date, the dynamics of intrinsically formed and exogenously seeded tau inclusions have not been extensively studied or compared. As different mechanisms of tau aggregate formation and different *MAPT* variants may differentially affect steady state tau levels, degradation and tau-induced neurotoxicity [31], we have developed and evaluated tau dynamics in both ‘intrinsic’ and ‘seeded’ brain slice culture (BSC) models of tau inclusion formation. In our previous work, we demonstrated that BSCs transduced with *MAPT* encoding the P301L/S320F double mutation [10, 50] develop an abundance of mature neurofibrillary pathology by 28 days in vitro (DIV). The tau inclusions that form are insoluble, Thioflavin S positive, and show fibrillar structure by electron microscopy (EM) [10]. Notably, BSCs are highly predictive of results obtained upon transgenic or rAAV-mediated expression of tau in rodent models [35].

We exploited the accessibility of BSCs for ex vivo imaging in combination with Dendra2-tagged rAAVs to examine the turnover of tau in living BSCs over an extended culture period. Tagging tau with photoswitchable Dendra2 provides a rapid technique to examine protein dynamics and stability without radioactivity or translation inhibitors required as in conventional pulse-chase studies [1, 6]. Here we sought to leverage optical pulse labeling experiments to observe tau inclusion formation dynamics and examine the kinetics of tau protein in different BSC models of tauopathies.

Using this experimental paradigm, we demonstrate that inclusions form in BSCs expressing P301L/S320F and do not form in BSCs expressing wild-type (WT) tau. However, P301L/S320F tau inclusions do demonstrate appreciable clearance with an average overall half-life of ~ 7 days that is approximately twice the half-life of the WT tau-Dendra2. This turnover is observed as both decreases in photoconverted species and replacement within the inclusion by more recently synthesized non-photoconverted tau. In our newly developed ‘seeded’ BSC model of tau aggregation, we find that P301L tau seeded with preformed K18 tau fibrils shows similar dynamics as observed in the intrinsic pro-aggregating tau BSC studies. Furthermore, upon aging in culture, these inclusions show increased half-lives demonstrating the applicability of this system to investigate altered tau kinetics. Notably, in both models, tau inclusions can be seen to form rapidly over a period of 12–96 h, and once filling the soma remain relatively constant in size with photoconverted tau eventually being replaced by non-photoconverted tau. These studies demonstrate that BSCs and optical pulse labeling methodology can be used to study tau protein dynamics and inclusion formation and reveal that tau inclusions form rapidly but then remain dynamic structures with appreciable daily turnover of tau within the inclusion. Together, this data provides powerful implications that targeting tau inclusion turnover therapeutically may form a novel line of treatment for tauopathies.

## Materials and methods

### rAAV production and preparation

Dendra2 plasmid (#51462) was obtained (Addgene, Watertown, MA, USA) and BamHI 5′ and MfeI 3′ sites were added by PCR. Dendra2 was removed and ligated into the CTR4 vector [10] to create pAAV-hCBA-Dendra2. To create the tau-Dendra2 constructs, previously created WT, P301L, P301L/S320F tau-EGFP constructs [10] were digested with the same restriction enzymes for Dendra2 to replace the EGFP gene. This was then ligated into the CTR4 vector using EcoRI and BamHI digests and BglII and MfeI digests on the tau-Dendra2 constructs, respectively. The MAP-2 variant of tau-Dendra2 was also cloned using the same strategy using a previously created MAP-2 AAV vector [10].

rAAV 2/8 expressing EGFP, 0N4R human tau; WT-tau, S320F-tau, P301L-tau, P301L/S320F-tau, Dendra2 [33], 0N4R human tau with the C-terminal Dendra2 tag; WT-tau-Dendra2, P301L-tau-Dendra2 and P301L/S320F-tau-Dendra2, under the control of the hybrid CBA promoter with CMV enhancer were generated as described previously [4, 5, 46]. P301L/S320F-tau-Dendra2 was also generated under the control of the MAP-2 promoter and packaged in rAAV 2/8-2Y as reported previously [10, 27]. rAAVs were applied to BSCs by adding rAAVs into the culture medium on the first day of culture (0 DIV) at 1–2 × 10^10^ VGs per well containing 3 BSCs.

### Expression, purification, assembly, and addition of recombinant K18 tau fibrils

WT K18 tau protein (the ~ 14 kDa microtubule binding domain (MTBD) in 4R human tau allowing the differentiation between overexpressed full length forms and truncated seeds) was prepared and fibrils were assembled as previously reported [50]. In brief, the cDNA corresponding to the human tau K18 fragment (residues Q244-E372 in 2N/4R human tau) with an added methionine residue at the N-terminus cloned in the bacterial expression plasmid pRK172 was expressed in BL21 (DE3)/RIL *Escherichia coli* (Agilent Technologies, Santa Clara, CA, USA) and was purified as previously described [22]. Protein concentrations were determined using a bicinchoninic acid (BCA) assay (Thermo Fisher Scientific, Waltham, MA, USA). Recombinant K18 tau protein (1 mg/ml) was assembled into filaments by incubation at 37 °C in sterile PBS with 50 μM heparin while shaking for at least 48 h. Tau fibril formation was confirmed by K114 or Thioflavin T fluorometry [50]. Heparin was removed from tau fibrils by centrifugation at 100,000×*g* and tau fibrils were resuspended in sterile PBS, with the resulting protein concentrations determined by BCA assay. Tau filaments were fragmented into shorter tau “seeds” by bath sonication for 60 min as previously reported [53]. In experiments where BSCs were seeded, 2 μg of K18 tau fibrils were applied directly on top of each BSC at DIV 14, as indicated in schematic diagrams (Figs. 6a, 7a, 9a, 9d).

### Organotypic brain slice cultures

All animal procedures were approved by the Institutional Animal Care and Use Committee at the University of Florida. BSCs were prepared from postnatal day 8 (p8) B6/C3H F1 mice (Envigo, Indianapolis, IN, USA) as previously reported [10, 14]. In brief, pups were cryoanesthetized, decapitated and the brains removed and dissected to retain the cortex, hippocampus and connecting regions in each hemi-brain in sterile filtered ice-cold dissection buffer [Hank’s balanced salt solution (HBSS), calcium, magnesium, no phenol red (Thermo Fisher Scientific), 2 mM ascorbic acid (Sigma Aldrich, St Louis, MO, USA), 39.4 μM ATP (Sigma Aldrich), and 1% (v/v) penicillin/streptomycin (Thermo Fisher Scientific)]. The hemi-brain was placed on filter paper and 350 µm coronal slices were cut using a McIllwain™ tissue chopper (Mickle Laboratory Engineering Co. Ltd., Surrey, UK). Slices were collected and plated randomly to contain three slices per semi-porous membrane insert (Millipore, 0.4 µm pore diameter, Thermo Fisher Scientific) in six-well sterile culture plates. Slices from different parts of the brain were distributed randomly across each well of every plate. Slices were maintained at 37 °C and 5% CO_2_ in sterile-filtered culture medium (basal medium eagle (BME, Thermo Fisher Scientific), 26.6 mM HEPES (pH 7.1, Thermo Fisher Scientific), 511 μM ascorbic acid, 1% (v/v) GlutaMAX (Thermo Fisher Scientific), 0.033% (v/v) insulin (Sigma Aldrich), 1% (v/v) penicillin/streptomycin (Thermo Fisher Scientific) and 25% (v/v) heat-inactivated horse serum (Sigma Aldrich). Culture medium was changed every 3–4 days. rAAVs were applied directly into the culture medium on the first day of culture (DIV 0) at 1–2 × 10^10^ genome particles of rAAV per well containing 3 BSCs.

### Sarkosyl extraction of BSCs

BSCs for assessment of insoluble tau from three wells (nine slices in total) were harvested into ice-cold phosphate buffered saline (PBS) and presented as *n* = 1. Sarkosyl-insoluble extractions were performed as previously described [10, 13]. In brief, the supernatant (S1) was kept and run on Western blots as the soluble fraction and the pellet (P3) was resuspended in 4 M Urea 2% SDS buffer and run on Western blots as the sarkosyl-insoluble fraction. Protein concentrations of S1 and P3 were determined by BCA assay before equal amounts of protein were resolved by SDS-PAGE and Western blotting.

### Antibodies

The following antibodies were used for Western blotting, immunohistochemistry and/or immuno-EM. A rabbit polyclonal antibody 3026 raised against full length recombinant 0N3R human tau [51], 94-3A6 (3A6), a mouse monoclonal antibody which recognizes the 4th MTBD of tau [13] and mouse monoclonal antibody 7F2 which recognizes tau phosphorylated at Thr 205 [51]. The following mouse monoclonal tau antibodies were kindly provided by Dr. Peter Davies (Feinstein Institute for Medical Research, Manhasset, NY, USA): CP13 (tau phosphorylated at Ser 202) and PHF-1 (tau phosphorylated at Ser 396 and Ser 404) [34]. Mouse monoclonal antibody to β-actin (Sigma Aldrich), was also used.

### SDS-PAGE and Western blotting

3–20 µg protein was separated on 4–12% (w/v) SDS-PAGE gels (Bio-Rad Laboratories, Hercules, CA, USA) and electrophoretically transferred to polyvinylidene difluoride (PVDF) membranes, as described previously [38]. Membranes were blocked in 0.5% casein for 1 h and then incubated with primary antibodies overnight at 4 °C, washed three times with TBS before incubation with fluorophore-conjugated Alexa Fluor 680 anti-mouse IgG (Thermo Fisher Scientific) or IRDye 800 goat anti-rabbit IgG (Li-Cor Biosciences, Lincoln, NE, USA) secondary antibodies and washed three times with TBS. Protein bands were detected and quantified using the multiplex Li-Cor Odyssey Infrared Imaging system (Li-Cor Biosciences).

### Immunohistochemistry and confocal imaging

BSCs were washed in PBS and then fixed on their inserts in 4% paraformaldehyde (PFA) for 1 h and stained as previously described [12]. In brief, individual BSCs (*n* = 1) were cut out from their membranes after fixation and then treated as free-floating sections for the following steps. BSCs were permeabilized and blocked for 18 h in 0.5% Triton X-100 in 20% bovine serum albumin (BSA, Sigma Aldrich) at 4 °C. BSCs were then incubated with appropriate primary antibodies overnight at 4 °C in 5% BSA, washed and then incubated with fluorophore-coupled secondary antibodies for 4 h at RT. Slice cultures were washed a final time before mounting on slides with Fluoromount-G (Southern Biotech, Birmingham, AL, USA) and then imaged using an Olympus FV1200 IX83 confocal laser-scanning microscope (Olympus America Inc, Center Valley, PA, USA). Z-stacks were captured over 15 µm at recommended step-sizes and projected as a maximum projection image using the Olympus Fluoview software.

### Thiazin Red staining

Organotypic BSCs were washed in PBS and then fixed on their inserts with 4% paraformaldehyde for 1 h. A stock solution of 1% Thiazin Red (Chemsavers Inc, Bluefield, VA, USA) in ddH_2_O was prepared and filtered through a 0.2-µm filter. Individual BSCs (*n* = 1) were cut from their membranes and autofluorescence reagent (Millipore) was applied for 5 min, and then washed in 40% ethanol (EtOH). BSCs were incubated with 0.05% Thiazin Red in ddH_2_O for 5 min in the dark, and then washed in 50% EtOH and then PBS. BSCs were mounted on slides with Fluoromount-G (Southern Biotech) and then confocal imaged as for immunohistochemistry to identify any amyloidogenic β-sheet structures in these sections.

For quantification of Dendra2 positive cells that were also Thiazin Red positive, images were captured on the Keyence all-in-one fluorescence microscope. Dendra2 expressing cells were automatically counted using the hybrid cell count feature and then the proportion that were also Thiazin Red were automatically quantified on the inbuilt Keyence analysis software.

### Long-term live imaging and quantification of Dendra2 in BSCs

Images of BSCs were captured using a Keyence BZ-X700 all-in-one fluorescence microscope (Keyence Corporation of America, Itasca, IL, USA). Dendra2 transduced BSCs were photoconverted using a 2 s pulse of blue light through a 20 µm Z-stack at recommended step-sizes with a 20 × objective lens. Images of emitted green and red fluorescence through a 20 µm Z-stack at recommended step-sizes were captured at the relevant time points and then projected onto a full focus image using the BZ-analyzer software (Keyence Corporation of America). Each red and green fluorescent image from each slice at each time point was captured from pre-determined regions. For population analysis, total corrected fluorescence was calculated using integrated density above background integrated density for each BSC using ImageJ (Version 1.51 k, National Institutes of Health, Bethesda, MD, USA) as previously reported [27]. For single cell analysis, a sample of individual living cells were manually identified from the last time frame over *n* = 5–6 BSCs prepared from different animals. Cells were aligned in ImageJ to correct for experimental drift over the time series [52], and fluorescent intensity for red and green channels were recorded for each cell. The median background fluorescence at each time frame was calculated and subtracted.

### EthD-1 cytotoxicity assay

Cytotoxicity was assessed by measuring EthD-1 uptake as previously described [10]. Three images of each slice were captured blindly from predetermined regions using a 10 × objective lens on a Keyence BZ-X700 all in one fluorescence microscope. EthD-1 positive cells were automatically counted using the hybrid cell count feature on the inbuilt Keyence analysis software, and the mean number of positive cells calculated from the three images captured.

### Immuno-electron microscopy

BSCs were washed in PBS and then fixed by 4% paraformaldehyde/0.25% glutaraldehyde in PBS for 1 h and processed for pre-embedding immuno-electron microscopy as previously described [21]. In brief, BSCs were incubated in 0.1% sodium borohydrate in PBS for 10 min and blocked with a solution of 5% horse serum, 1% bovine serum albumin, and 0.2% cold water fish skin gelatin in PBS for 1 h. BSCs were incubated overnight at 4 °C with primary antibody—7F2 to tau phosphorylated at Thr205 [51]. BSCs were then incubated with biotinylated-conjugated goat anti-mouse secondary antibody and developed with diaminobenzidine using Vectastain ABC kit as previously reported [56]. Tissue was silver enhanced as described by Rodriguez [45] and then post-fixed with 2% glutaraldehyde and 2% osmium tetraoxide, and, following dehydration in graded EtOH, they were embedded in Epon. Ultrathin sections of BSCs were cut and then negatively stained with 1% uranyl acetate and visualized with a Hitachi 7600 transmission electron microscope.

### Statistical analysis

Data were analyzed statistically according to the methods specifically referred to in each figure legend. Data were compared by Student’s two-tailed unpaired *T* test, one-way analysis of variance (ANOVA) with post hoc Tukey’s multiple comparisons test or two-way ANOVA with post hoc Sidak’s test (Graphpad Prism 8.0 Software, La Jolla, CA, USA). Half-lives were calculated using non-linear regression analysis of one-phase decay. A two-sided Kolomogorov–Smirnov test was used to determine differences between probability distributions. Differences were considered statistically significant when *p* < 0.05. All graphs were generated in GraphPad Prism.

## Results

### Pro-aggregant P301L/S320F-tau-Dendra2 forms authentic neurofibrillary inclusions in BSCs

rAAV-mediated expression of human P301L/S320F-tau induces rapid and robust fibrillar tau pathology in BSCs [10]. Dendra2 has been used in previous studies to identify localized distributions of tau [39], but we sought here to exploit the photoswitchable properties of Dendra2 [6, 32] to gain insight to the dynamics underlying soluble and aggregated tau protein. We generated rAAVs of Dendra2 alone, and Dendra2 fused to the C-terminus of human 4R0N WT tau and human 4R0N P301L/S320F tau. Dendra2 is a photoswitchable fluorescent protein that emits only green fluorescence until it is exposed to short-wavelength light, which induces an irreversible conformation change to switch their spectral properties to emit red fluorescence and then reduce total green fluorescence [7] Any proteins synthesized post-conversion will emit green fluorescence above the residual green fluorescence allowing assessment of protein dynamics using long-term live microscopy [6, 32].

**Fig. 1:**
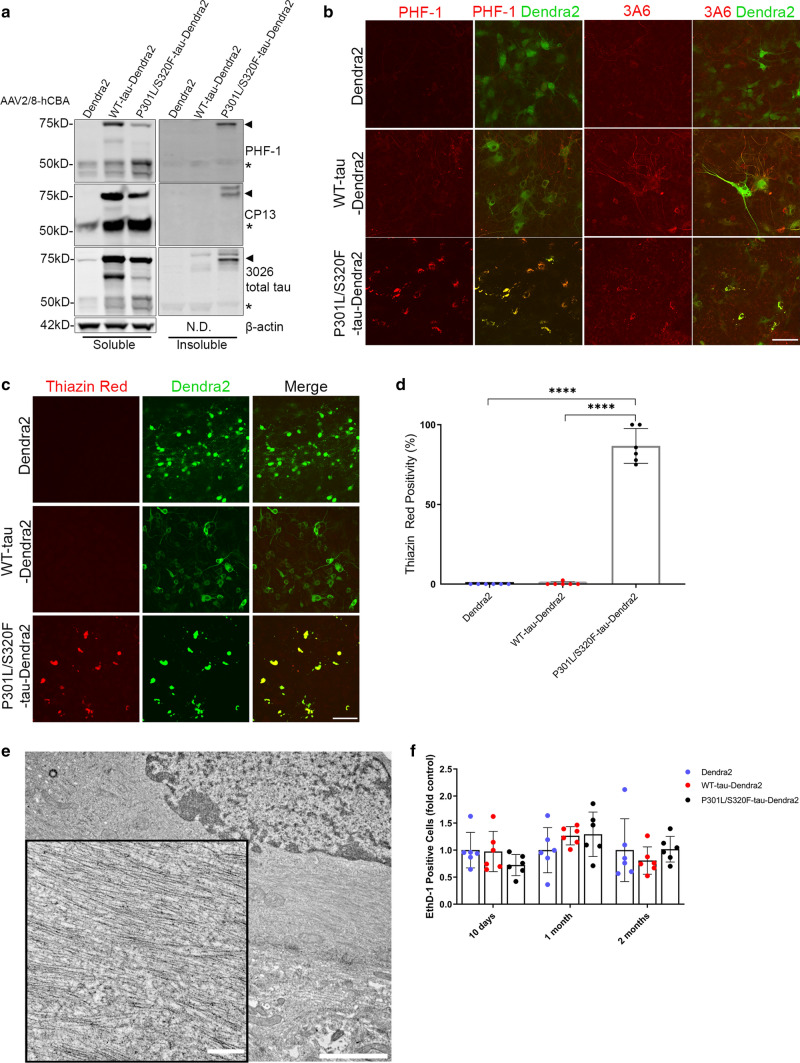
P301L/S320F-Tau-Dendra2 BSCs develop mature neurofibrillary tau inclusions. BSCs were transduced at 0 DIV with rAAVs to express Dendra2, WT-tau-Dendra2 or P301L/S320F-tau-Dendra2 and maintained in culture until 10 DIV. **a** BSCs were sequentially extracted to prepare soluble and sarkosyl-insoluble fractions. Lysates were analyzed on western blots for tau phosphorylated at Ser202 (CP13), tau phosphorylated at Ser396/404 (PHF-1), total tau (3026) and β-actin as a loading control. Representative western blots of the soluble and sarkosyl-insoluble fractions are shown (*n* = 3). The mobility of molecular mass markers are shown on the left. Black arrows indicate tau-Dendra2 fusions, asterisks indicate endogenous tau and absence of Dendra2 tag cleavage. **b** BSCs were fixed, immunostained for PHF-1 and total tau (3A6) and confocal imaged to confirm the distribution of tau. Scale bar = 25 µm (*n* = 3). **c** Transduced BSCs were fixed and stained with Thiazin Red to identify any β-sheet structures in these sections. Scale bar = 50 µm (*n* = 6). **d** Bar graph shows quantification of proportion of cells in transduced BSCs that expressed Dendra2 and were Thiazin Red positive. Data are mean ± SEM (*n* = 6). **e** P301L/S320F-tau-Dendra2 transduced BSCs were fixed and examined by immuno-EM for the presence of filamentous tau-positive inclusions. Immuno-labeling with antibody against tau phosphorylated at Thr205 (7F2) is shown. Enlarged section shows fibrillar tau. Scale bars; 0.2 µm (right); 200 nm (left, enlargement). **f** Cell death in BSCs at 10DIV, 1 month and 2 months in culture was assessed by EthD-1 uptake. Bar graph shows quantification of EthD-1-positive cells as a proportion of Dendra2 (control). Data are mean ± SEM (*n* = 6)

We characterized the development of tau pathology in BSCs that were transduced with Dendra2, WT-tau-Dendra2 and P301L/S320F-tau-Dendra2 beginning at 10 DIV, as a large proportion of cells expressing P301L/S320F-tau-Dendra2 already bear tau inclusions. Biochemical assessments [13] identify overexpression of total tau (3026) [51] in BSCs expressing WT-tau-Dendra2 and P301L/S320F-tau-Dendra2, as well as, accumulation of soluble tau phosphorylated at Ser202 (CP13) and Ser396/404 (PHF-1) (Fig. 1a). BSCs expressing P301L/S320F-tau-Dendra2 developed sarkosyl-insoluble, hyperphosphorylated tau that was not detected in BSCs expressing Dendra2 or WT-tau-Dendra2. We also identified no increases in 50 kDa tau, which may occur if the Dendra2 tag was being cleaved from tau (Fig. 1a). We also examined the distribution of tau inclusion pathology in BSCs by immunohistochemistry (Fig. 1b). Both WT-tau-Dendra2 and P301L/S320F-tau-Dendra2 transduced BSCs show overexpression of tau as detected by 3A6 staining [13], with WT-tau-Dendra2 transduced BSCs developing Ser396/404 phosphorylated tau throughout the cytoplasm of neurons. In contrast, P301L/S320F-tau-Dendra2 transduced BSCs show Ser396/404 phosphorylated tau exclusively in the soma by 10 DIV.

We examined fibrillar tau pathology by staining tau-Dendra2 transduced BSCs with the dye Thiazin Red which recognizes NFTs in human AD [40, 41]. BSCs transduced with P301L/S320F-tau-Dendra2 develop aggregates that are Thiazin red positive, with staining absent in BSCs transduced with Dendra2 or WT-tau-Dendra2 (Fig. 1c). Quantification identified that 86.72 ± 4.45% (mean ± SEM) of P301L/S320F-tau-Dendra2 cells are Thiazin Red positive (Fig. 1d). We further studied P301L/S320F-tau-Dendra2 transduced BSCs by immuno-electron microscopy (EM) with the 7F2 antibody to tau phosphorylated at Thr205 [51] (Fig. 1e). These studies show that fibrillar tau accumulates in these cultures comparable to those we identified in BSCs transduced with untagged pro-aggregant tau [10] and those found in human AD [15, 29]. We also evaluated cytotoxicity in BSCs expressing Dendra2, WT-tau-Dendra2, P301L/S320F-tau-Dendra2 by an EthD-1 uptake assay at 10 DIV, 1 month and 2 months in culture and identified no increased toxicity at any of these time points compared to the Dendra2 control (Fig. 1f) similar to our previous findings using untagged human tau rAAVs [10].

These data confirm that tau pathology with a Dendra2 tag recapitulates previous studies using tau or tau-EGFP transduced BSCs, with P301L/S320F-tau-Dendra2 transduced BSCs developing mature neurofibrillary pathology by DIV 10 [10]. Indeed, after 10 DIV, we have established that the majority of inclusions within cells in the rAAV P301L/S320F-tau-Dendra2 transduced BSCs are fibrillar aggregates.

### Optical pulse labeling methodology reveals P301L/S320F-tau-Dendra2 shows turnover in BSCs

**Fig. 2:**
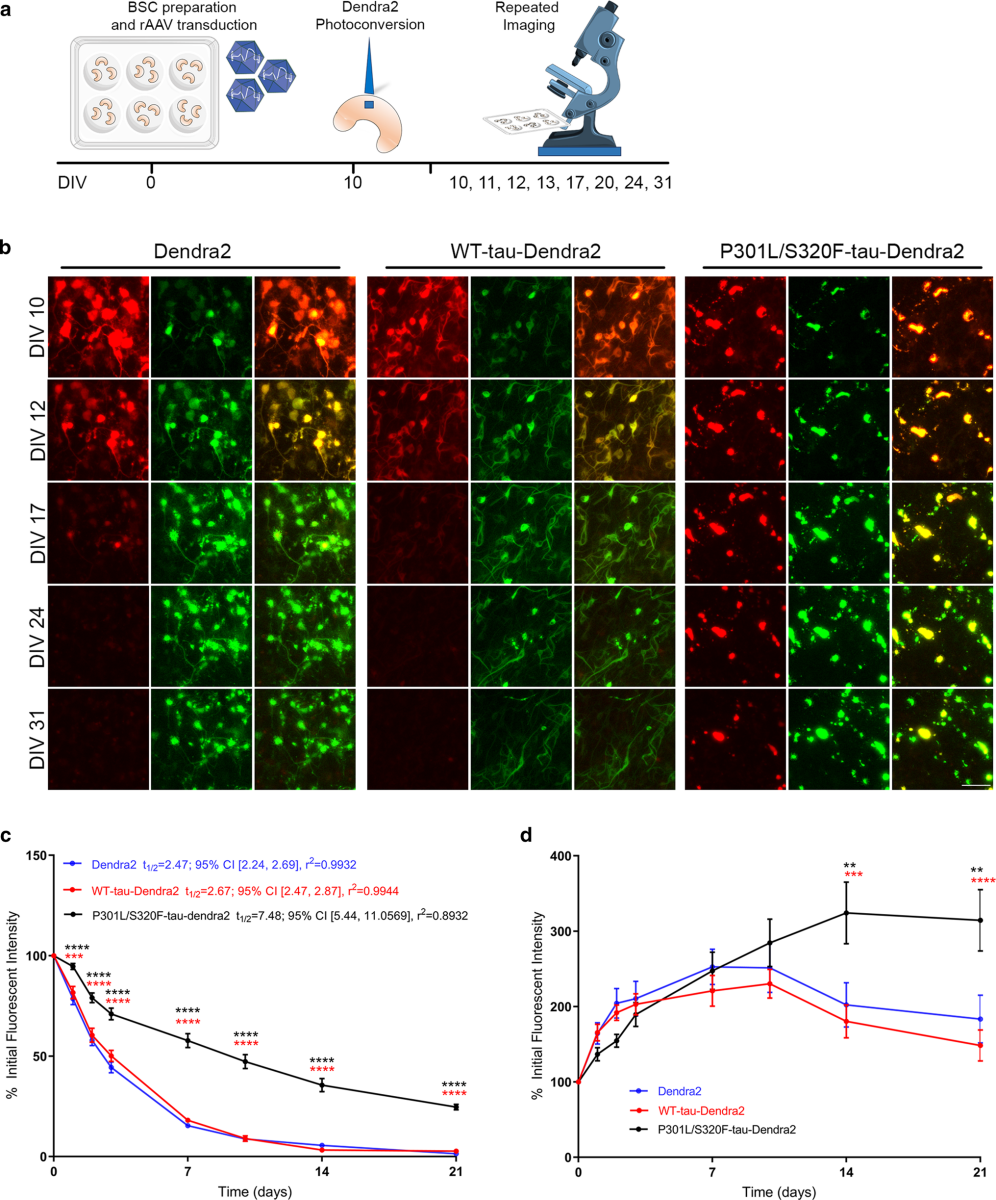
P301L/S320F-tau-Dendra2 is long-lived compared to WT-tau-Dendra2 but is produced at a similar rate in BSCs. **a** A schematic diagram shows the timeline of the long-term optical pulse labeling experiments using photoconversion of Dendra2, WT-tau-Dendra2 and P301L/S320F-tau-Dendra2 over 31 DIV. **b** Representative images of emitted fluorescence of photoconverted (red) and newly synthesized (green) Dendra2 in BSCs transduced at 0 DIV with Dendra2, WT-tau-Dendra2, or P301L/S320F-tau-Dendra2, photoconverted at 10 DIV and imaged at several time points from 10 DIV (immediately post-photoconversion) until 31 DIV (21 days post-photoconversion). Merge of both channels is also shown. Scale bar = 50 µm. **c** Line graphs show amounts of photoconverted Dendra2 fluorescent intensity in each condition that were quantified over time as a proportion of initial fluorescent intensity (*n* = 12, data are mean ± SEM, analyzed by Two-way ANOVA with post hoc Sidak’s test; ***p < 0.001, ****p < 0.0001, *p* values in black; Dendra2 v P301L/S320F-tau-Dendra2, *p* values in red; WT-tau-Dendra2 v P301L/S320F-tau-Dendra2). **d** Line graphs show amounts of green Dendra2 fluorescent intensity in each condition that were quantified over time as a proportion of initial fluorescent intensity (*n* = 12, data are mean ± SEM, analyzed by two-way ANOVA with post hoc Sidak’s test; **p < 0.01, ***p < 0.001, ****p < 0.0001, *p* values in black; Dendra2 v P301L/S320F-tau-Dendra2, *p* values in red; WT-tau-Dendra2 v P301L/S320F-tau-Dendra2)

The precise kinetics and stability of tau inclusions bearing aggregated tau and their relationship to neurodegeneration remains to be determined. Here we examined the synthesis and turnover of WT tau compared to pro-aggregant P301L/S320F tau by executing long-term optical pulse labeling experiments to estimate the turnover of tau in these conditions. A schematic diagram of the long-term imaging timeline is shown in Fig. 2a. We tracked the red fluorescent photoconverted Dendra2-tagged tau pool using long-term imaging from 10 to 31 DIV, as well as newly synthesized non-photoconverted green fluorescent Dendra-2 tagged tau (Fig. 2b). Upon population level quantification, amounts of red photoconverted P301L/S320F-tau-Dendra2 was significantly higher than Dendra2 or WT-tau-Dendra2 throughout the long-term imaging study from 1 to 21 days post photo-conversion (Fig. 2c). When estimating half-lives from this data; Dendra2 alone shows a half-life of 2.47 days; 95% CI [2.24, 2.69], *r*^2^ = 0.99, WT-tau a half-life of 2.67 days; 95% CI [2.47, 2.87], *r*^2^ = 0.99 and P301L/S320F-tau shows a significantly longer half-life of 7.48 days; 95% CI [5.44, 11.06], *r*^2^ = 0.89. These results indicate that WT tau turns over in the same time frame as Dendra2 alone; whereas P301L/S320F-Dendra2 pro-aggregant tau is more stable but does decline over time albeit with a longer half-life compared to Dendra2 or WT-tau-Dendra2, suggesting a slower but apparent turnover. We also confirmed that the half-life of Dendra2 does not significantly depend on initial fluorescent intensity or expression levels (Supp. Figure 1), as per previous reports [1].

It is plausible that aggregated tau may accumulate in BSCs and in disease due to a higher overall production rate of tau. Previous studies have suggested that some MAPT mutations may increase overall tau production in tau transgenic mice [54, 55]. In human AD patients, CSF tau is also produced at a higher rate than controls [49]. We therefore tracked the production of new pools of WT and P301L/S320F tau by quantifying total green fluorescence which would be expected to increase over time due to protein synthesis. After photoconversion at 10 DIV, we imaged and quantified the newly produced green non-photoconverted tau pool using long-term live imaging until 31 DIV (Fig. 2b, d). Dendra2 alone, WT tau and P301L/S320F tau all increased at a similar rate, with newly synthesized P301L/S320F tau significantly accumulating more at 14 DIV after the initial photoconversion (as predicted by the slower decline of red photoconverted tau), and WT tau and Dendra2 showing a faster turnover of the newly synthesized protein at these long-term imaging periods.

**Fig. 3:**
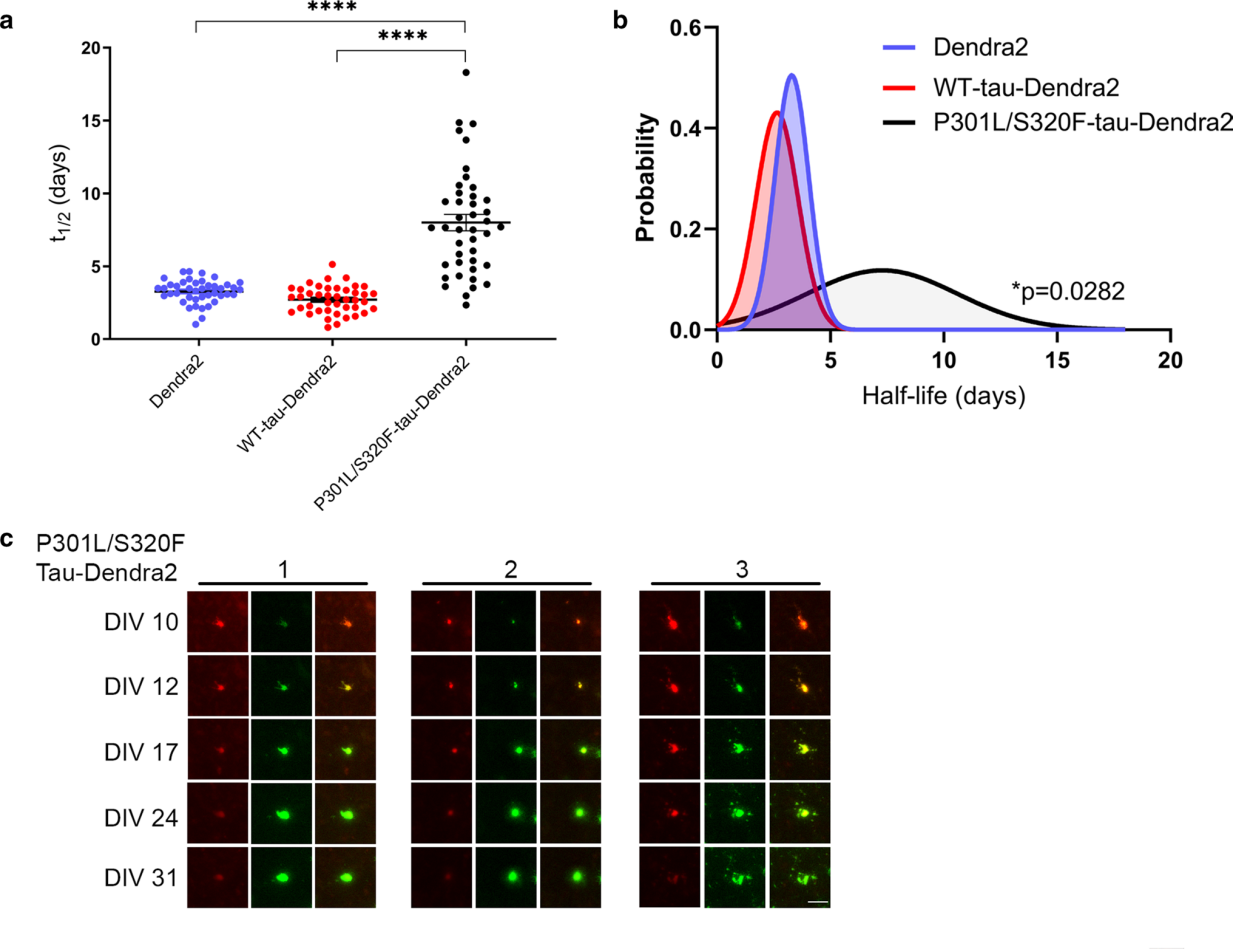
Clearance and decline in P301L/S320F-tau-Dendra2 is partially attributed to turnover of inclusions. **a** Scatter plot of half-life estimates for individual living cells in BSCs expressing Dendra2, WT-tau-Dendra2 or P301L/S320F-tau-Dendra2 (*n* = 42; ****p < 0.0001, one-way ANOVA). **b** Probability density plot of half-life estimates for individual living cells expressing Dendra2, WT-tau-Dendra2 or P301L/S320F-tau-Dendra2 (*n* = 42; *p = 0.0282, two-sided Kolmogorov–Smirnov test of P301L/S320F-tau-Dendra2 v Dendra2/WT-tau-Dendra2). Values were pooled from 6 BSCs per condition. **c** Representative images of three examples of P301L-S320F-Dendra2-expressing cells which show turnover of photoconverted (red) tau in inclusions while newly synthesized (green) fluorescence demonstrates the cell is alive. Merge showing exchange of red and green P301L/S320F-tau within individual cells is also shown. Scale bar = 25 µm

To examine the dynamics of tau turnover, we further evaluated, at an individual cellular level, the rate of tau turnover and replacement in BSCs. For this analysis, a sample of individual cells alive at 31 DIV were selected as any cell death could bias half-life calculations. We also demonstrated earlier no significant levels of toxicity above Dendra2 control up to 2 months in culture. This analysis of living individual cells identified the mean half-life of P301L-S320F-tau-Dendra2 as 7.99 days; 95% CI [6.87, 9.12], compared to Dendra2 at 3.27 days; 95% CI [3.02, 3.51] and WT-tau-Dendra2 at 2.70 days; 95% CI [2.41, 2.99] (Fig. 3a). We also performed a probability density analysis, confirming the P301L/S320F tau shows a significantly longer half-life (Fig. 3b). Representative images of individual inclusions turning over in pro-aggregant P301L/S320F-Dendra2 BSCs are shown in Fig. 3c. In many cases, the red photoconverted tau in the soma, decreased over time and was simply replaced by green fluorescence. If the amount of red fluorescence was relatively small, then this was often completely replaced by green fluorescence within 21 days. In contrast, larger initial levels of red cytoplasmic fluorescence, would show decreasing red fluorescence and replacement by green fluorescence, increasingly over 7–21 days. However, the overall size of the fluorescent ‘inclusion’ appears to remain consistent or only slightly increased.

Overall, by characterizing and then using optical pulse labeling methods in BSCs, we have identified that once a tau inclusion is formed, it can turn over and is replaced by newly synthesized pools of tau whilst the overall size of the inclusion is restricted.

### Extended time in culture increases the half-life of P301L/S320F-tau-Dendra2 inclusions

**Fig. 4:**
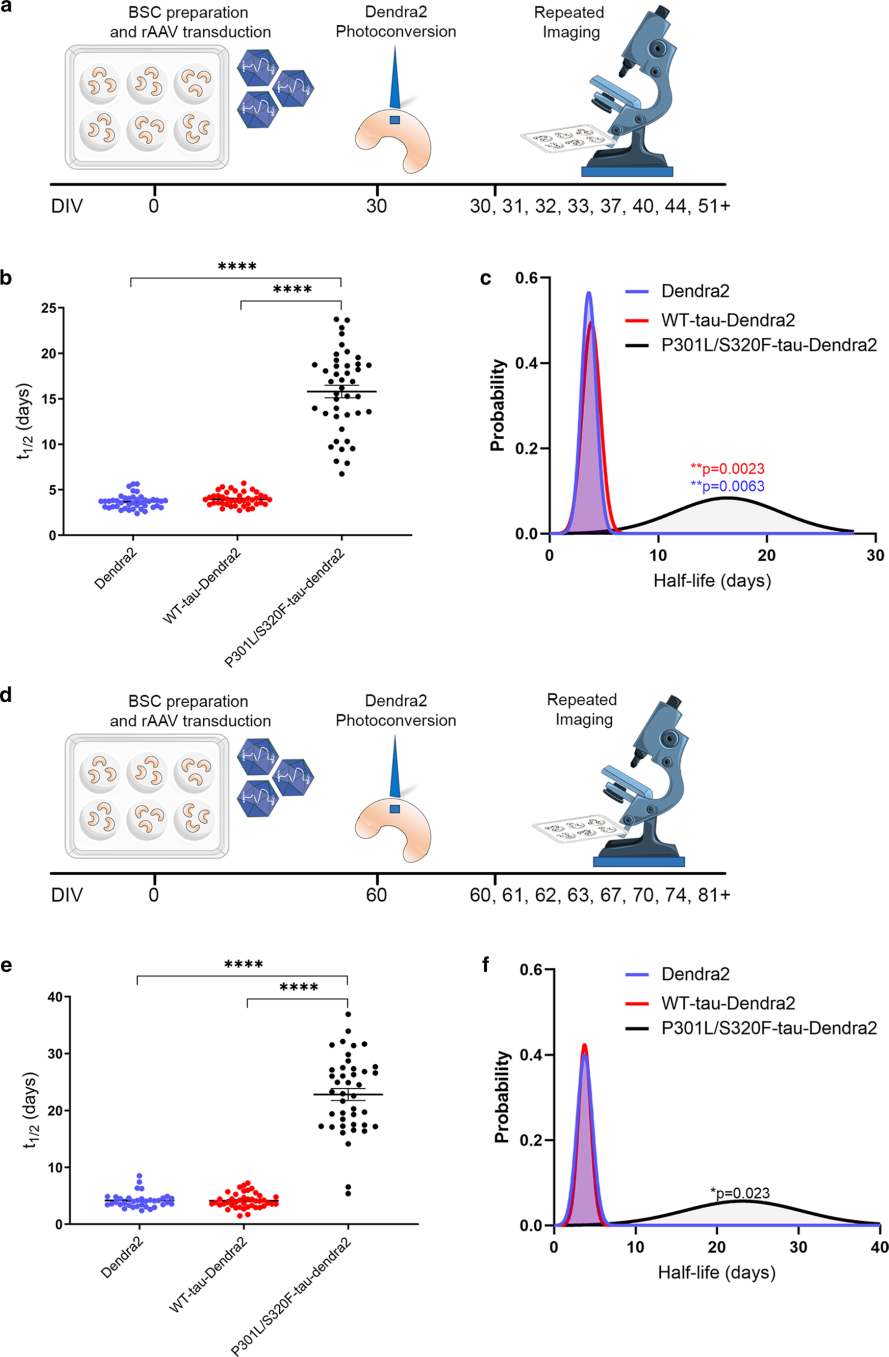
Tau inclusions continue to turnover after extended periods in culture but at a slower rate. **a** Schematic diagram shows the timeline of the long-term optical pulse labeling experiments using photoconversion of Dendra2, WT-tau-Dendra2 and P301L/S320F-tau-Dendra2 imaging photoconverting at a later time point in culture ~ 1 month old. **b** Scatter plot of half-life estimates for individual living cells in BSCs expressing Dendra2, WT-tau-Dendra2 or P301L/S320F-tau-Dendra2 (*n* = 42; ****p < 0.0001, one-way ANOVA). **c** Probability density plot of half-life estimates for individual living cells expressing Dendra2, WT-tau-Dendra2 or P301L/S320F-tau-Dendra2 (*n* = 42; **p = 0.0023, two-sided Kolmogorov–Smirnov test of P301L/S320F-tau-Dendra2 v WT-tau-Dendra2, **p = 0.0063, two-sided Kolmogorov–Smirnov test of P301L/S320F-tau-Dendra2 v Dendra2). Values were pooled from 6 BSCs per condition. **d** Schematic diagram shows the timeline of the long-term optical pulse labeling experiments using photoconversion of Dendra2, WT-tau-Dendra2 and P301L/S320F-tau-Dendra2 imaging photoconverting at a further extended time point in culture ~ 2 months old. **e** Scatter plot of half-life estimates for individual living cells in BSCs expressing Dendra2, WT-tau-Dendra2 or P301L/S320F-tau-Dendra2 (*n* = 42; ****p < 0.0001, one-way ANOVA). **f** Probability density plot of half-life estimates for individual living cells expressing Dendra2, WT-tau-Dendra2 or P301L/S320F-tau-Dendra2 (*n* = 35–42; *p = 0.023, two-sided Kolmogorov–Smirnov test of P301L/S320F-tau-Dendra2 v Dendra2/WT-tau-Dendra2). Values were pooled from 5–6 BSCs per condition

After identifying that tau inclusions that were formed after 10 DIV turned over with a half-life of ~ 1 week, we sought to identify how ageing or duration in culture may affect half-life and turnover of these inclusions. A schematic diagram of the long-term imaging timeline is shown in Fig. 4a. We tracked the red fluorescent photoconverted Dendra2-tagged tau pool using long-term live imaging after photoconverting at ~ 1 month in culture, as well as newly synthesized non-photoconverted green fluorescent Dendra-2 tagged tau.

As for earlier analysis, we examined at an individual cellular level, the rate of tau turnover and replacement in BSCs that had been aged for ~ 1 month prior to photoconversion. This analysis of living individual cells identified the mean half-life of P301L-S320F-tau-Dendra2 as 15.80 days; 95% CI [14.42, 17.18], compared to Dendra2 at 3.67 days; 95% CI [3.44, 3.91] and WT-tau-Dendra2 at 3.97 days; 95% CI [3.74, 4.19] (Fig. 4b) after 1 month of aging. We also performed a probability density analysis, confirming the P301L/S320F tau shows a significantly longer half-life (Fig. 4c).

We then extended the time in culture prior to photoconversion and long-term imaging to ~ 2 months—a schematic diagram of this timeline is shown in Fig. 4d. We then quantified the rate of tau turnover and replacement in living cells in BSCs that had been aged for ~ 2 months prior to photoconversion. We identified the mean half-life of P301L-S320F-tau-Dendra2 after ~ 2 months of aging, as 22.80 days; 95% CI [20.68, 24.93], compared to Dendra2 at 4.17 days; 95% CI [3.72, 4.62] and WT-tau-Dendra2 at 4.13 days; 95% CI [3.72, 4.54] (Fig. 4e). Probability density analysis confirmed the P301L/S320F tau shows a significantly longer half-life (Fig. 4f).

Taken together, these findings suggest that extended culture periods or exposure to a tau inclusion increases the half-life of the inclusion turnover to ~ 2 weeks after 1 month of aging, and to ~ 3 weeks after 2 months of aging. This highlights the utility of this system for studying differing tau kinetics with age or different culture conditions.

### Development of a seeded BSC model of tau inclusion pathology

**Fig. 5:**
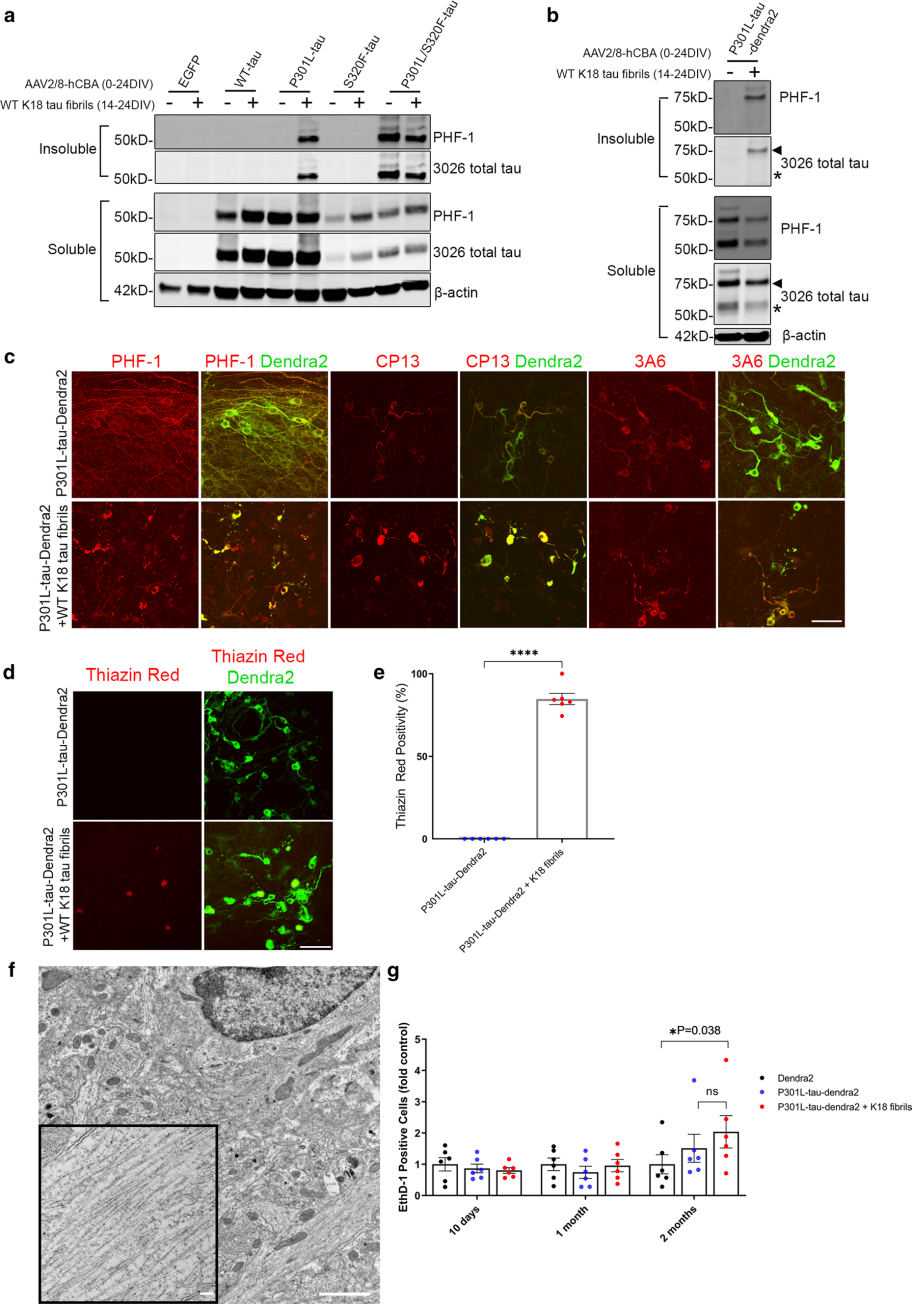
Seeding of P301L-tau-transduced BSCs induces phosphorylated and sarkosyl-insoluble tau inclusions. BSCs were prepared and transduced with rAAVs to express EGFP, WT-tau, S320F-tau, P301L-tau, P301L-tau-Dendra2 or P301L/S320F-tau at 0 DIV and then maintained in culture until 14 DIV at which point K18 tau fibrils were added. At 24 DIV BSCs were harvested and sequentially extracted to prepare soluble and sarkosyl-insoluble fractions. Lysates were analyzed by western blotting for tau phosphorylated at Ser396/404 (PHF-1), total tau (3026) and β-actin as a loading control. **a** Representative western blots from transduced BSCs in the soluble and sarkosyl-insoluble fraction are shown (*n* = 2). The mobility of molecular mass markers are shown on the left. **b** Representative western blots of BSC lysates from P301L-tau-Dendra2 with and without the addition of K18 tau fibrils in the soluble and sarkosyl-insoluble fraction are also shown. Black arrows indicate tau-Dendra2 fusions, asterisks indicate endogenous tau and absence of Dendra2 tag cleavage (*n* = 4). **c** P301L-tau-Dendra2-transduced BSCs with and without K18 tau fibril seeding were fixed, immunostained for PHF-1, CP13 and total tau (3A6) and confocal imaged to confirm the distribution of tau. Scale bar = 50 µm (*n* = 3). **d** Seeded and non-seeded P301L-tau-Dendra2 transduced BSCs were stained with Thiazin Red to identify amyloidogenic structures in these sections. Scale bar = 50 µm. **e** Bar graph shows quantification of proportion of cells in transduced BSCs that expressed Dendra2 and were Thiazin Red positive. Data are mean ± SEM (*n* = 6). **f** P301L-tau-Dendra2 transduced BSCs with K18 tau fibril seeding were fixed and prepared for EM. Immuno-labeling with 7F2 antibody to tau phosphorylated at Thr205 is shown. Enlarged section shows bundles of tau filaments. Scale bars; 0.2 µm (right); 100 nm (left, enlargement). **g** Cell death in BSCs at 10DIV, 1 month and 2 months post seeding in culture was assessed by EthD-1 uptake. Bar graph shows quantification of EthD-1-positive cells as a proportion of Dendra2 (control). Data are mean ± SEM (*n* = 6)

Tau pathology progresses spatiotemporally after disease onset in AD [2] and the seeding and propagation of tau inclusions has been identified as a possible mechanism for the spread of tau pathology in tauopathies [23]. To examine aggregation triggered by the addition of exogenous K18 tau preformed fibrils, we expressed different human tau variants in BSCs with and without fibrils. Biochemical analysis revealed that P301L 4R0N human tau can be robustly seeded in BSCs by the addition of exogenous K18 tau fibrils and phosphorylated insoluble tau accumulates (Fig. 5a). BSCs transduced with an EGFP control, WT or S320F tau and seeded with K18 tau fibrils only accumulated soluble phosphorylated tau (Fig. 5a). P301L/S320F tau seeded with K18 tau fibrils did not accumulate any further insoluble or phosphorylated tau (Fig. 5a). This data highlights the versatility of this platform to evaluate tau propagation and seeding.

To facilitate optical pulse labeling experiments of this seeded model, we generated rAAVs of Dendra2 fused to the C-terminus of human 4R0N P301L tau. We then biochemically characterized BSCs expressing P301L-tau-Dendra2 with and without K18 tau fibrils at the time point we planned to perform live imaging. P301L-tau-Dendra2 transduced BSCs were robustly seeded by K18 tau fibrils, showing the accumulation of insoluble, phosphorylated tau (Fig. 5b). We also confirmed no increased 50 kDa tau which could occur if the Dendra2 tag was being cleaved off.

We also performed immunohistochemistry to identify the distribution of inclusion pathology (Fig. 5c). Both P301L-tau-Dendra2 seeded and non-seeded BSCs show overexpression of tau [13]. P301L-tau-Dendra2 BSCs without the addition of exogenous K18 tau fibrils develop phosphorylated tau throughout the cytoplasm of neurons, whilst P301L-tau-Dendra2 BSCs that were seeded with K18 tau fibrils show accumulation of this pathology almost exclusively in the soma. To identify any neurofibrillary pathology, we stained with Thiazin Red and identified seeded P301L-tau-Dendra2 BSCs develop aggregates that are Thiazin red positive, with staining absent in BSCs transduced with P301L-tau-Dendra2 without the addition of fibrils (Fig. 5d). Quantification revealed at 10 days post-seeding with K18 fibrils 84.77 ± 3.41% of P301L-Dendra2 expressing cells were also Thiazin Red positive (Fig. 5e). Upon further examination by immuno-EM (Fig. 5f), seeded P301L-tau-Dendra2 BSCs accumulate tau filaments that are 7F2 positive. We also evaluated cytotoxicity in BSCs expressing P301L-tau-Dendra2 with and without exogenous K18 tau fibrils by an EthD-1 uptake assay at 10 DIV, 1 month and 2 months in culture post the addition of fibrils. We identified some increased toxicity in the seeded P301L-tau-Dendra2 BSCs 2 months post addition of seeds compared to the Dendra2 control (Fig. 5g).

These data confirm that P301L tau BSCs can be seeded with exogenous K18 tau fibrils to develop mature neurofibrillary pathology and provide a novel three-dimensional model of ‘seeded’ tau aggregation.

### Tau inclusions form rapidly in both intrinsic and ‘seeded’ BSC models

**Fig. 6:**
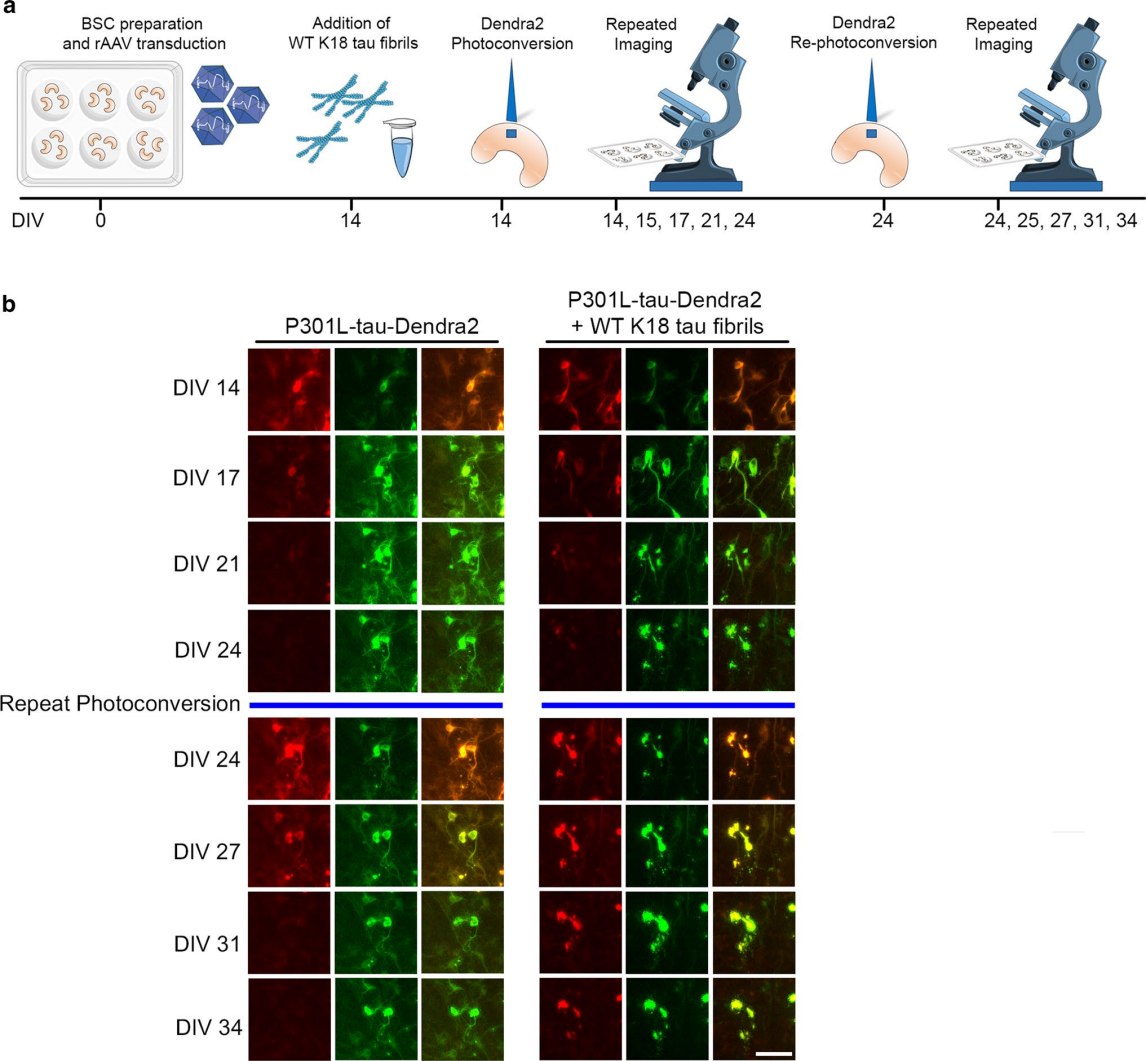
Inclusions form rapidly from soluble P301L-tau-Dendra2 when seeded and remain stable. **a** A schematic diagram shows the timeline of the long-term optical pulse labeling experiments using photoconversion of P301L-tau-Dendra2 with and without K18 tau fibril seeding over 34 DIV. BSCs were prepared and transduced with rAAVs to express P301L-tau-Dendra2 on 0 days in vitro (DIV) and then maintained in culture until 14 DIV before addition of K18 tau fibrils, photoconversion of Dendra2 and repeated imaging until 24 DIV, BSCs underwent a second photoconversion at 24DIV and then further imaging until 34 DIV to track formation and stability of inclusions. (b) Representative images of photoconverted (red) Dendra2, newly synthesized (green) Dendra2 and merge of channels are shown during the first 10 days with and without addition of K18 tau fibrils and after a second photoconversion at 24 DIV and further 10 days of imaging until 34 DIV to track the formation and stability of seeded inclusions. Scale bar = 50 µm

One of the main advantages of using ex vivo BSC models is their accessibility and ease for long-term imaging studies. As we identified that P301L-tau-Dendra2 tau can be induced to aggregate with the addition of exogenous K18 tau fibrils, we established a long-term imaging paradigm to track the formation and fate of inclusions in this model following addition of seeds. A schematic diagram of the imaging timeline to track inclusion formation with the addition of seeds is shown in Fig. 6a. P301L-tau-Dendra2 BSCs that were not seeded show turnover of tau after the initial and second repeated photoconversion suggesting a constant dynamic pool of tau turnover in this model (Fig. 6b). In P301L-tau-Dendra2 BSCs that undergo seeding with exogenous K18 tau fibrils, tau inclusions can be seen forming from both newly synthesized green fluorescent tau and initially photoconverted red fluorescent tau rapidly in the 10 day period after addition of seeds. When photoconverted for a second time, these inclusions remain more stable than the unseeded condition for the additional 10 day imaging period, highlighting that these stable aggregates rapidly formed in the previous 10 day period and may turnover more slowly.

To alternatively visualize and track tau inclusion formation, we used a MAP-2 promoter to restrict expression of P301L/S320F-tau-Dendra2 within neurons in BSCs. We have previously confirmed that tau inclusion pathology is driven in neurons using neuronal-specific promoters [10]. MAP-2 driven P301L/S320F-tau-Dendra2 expression resulted in Thiazin Red positive inclusions in BSCs like those observed when tau is expressed under the hCBA promoter (Supp. Figure 2a). Furthermore, as tau expression from the MAP-2 promoter is slower, this paradigm can be used to image the formation of neuronal tau inclusions over time (Supp. Figure 2b). Imaging P301L/S320F-tau-Dendra2 in BSC neurons showed that inclusions can form rapidly in less than 24 h or more slowly over a period of 96 h (Supp. Figure 2b) similar to those forming rapidly in seeded P301L-tau-Dendra2 BSCs and in vivo in tau transgenic mice [18]. Once formed, these inclusions also exist in culture for at least a further 7 DIV similar to our earlier findings using hCBA promoter.

Taken together, these findings in both ‘seeded’ and ‘intrinsic’ tau inclusion BSC models show that this system can be used to visualize the rapid formation of inclusions in neurons over a period 12–96 h, and their fate can continue to be tracked long term.

### Seeded P301L-tau-Dendra2 inclusions also show appreciable turnover of tau

**Fig. 7:**
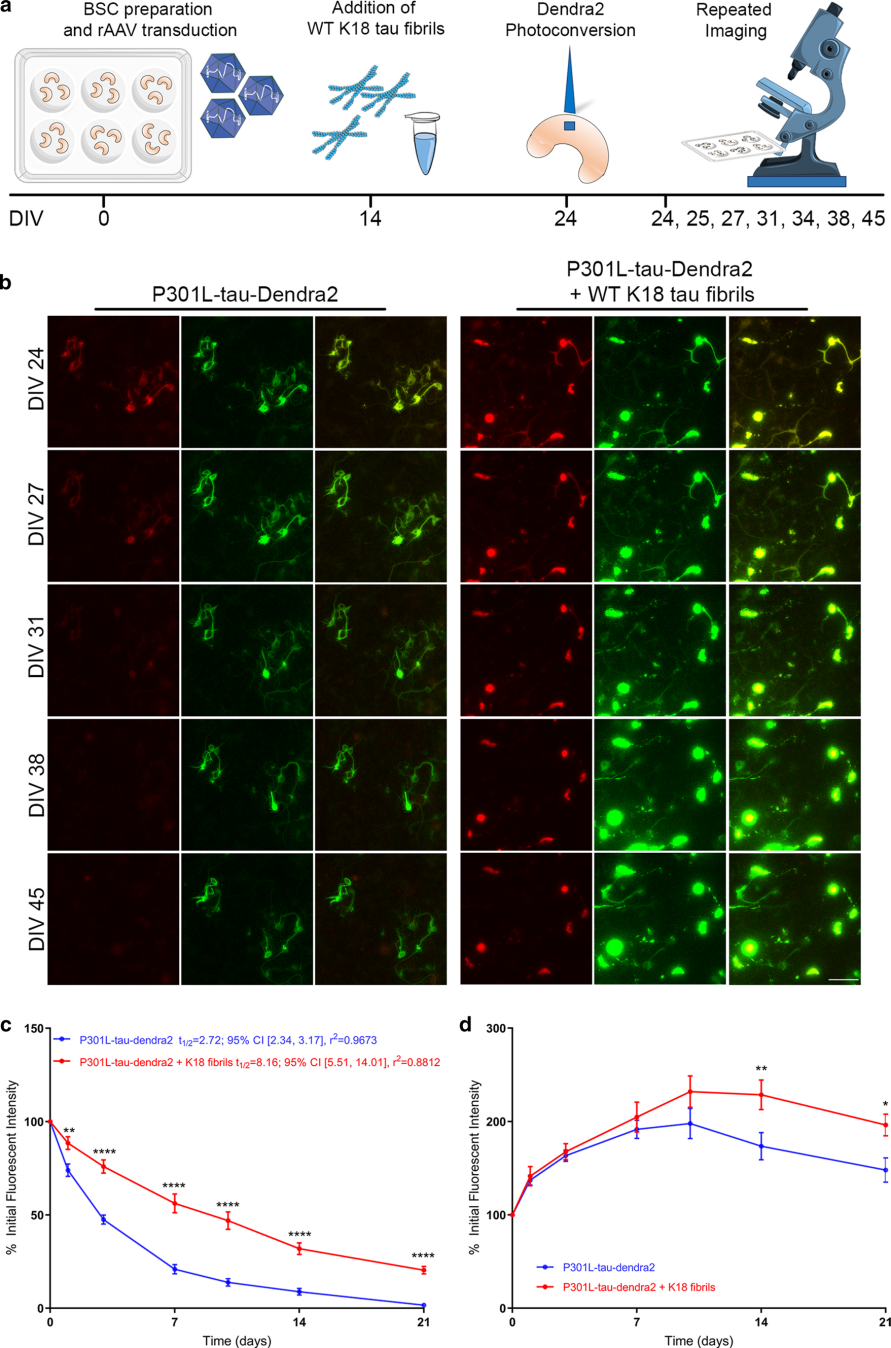
Seeding of P301L-tau-Dendra2 forms long-lived inclusions but does not affect tau production in BSCs. **a** A schematic diagram shows the timeline of the long-term optical pulse labeling experiments using photoconversion of P301L-tau-Dendra2 with and without K18 tau fibril seeding over 45 DIV. **b** Representative images of emitted fluorescence of photoconverted (red) and newly synthesized (green) Dendra2 from BSCs transduced at 0 DIV with P301L-tau-Dendra2 and then untreated or seeded with K18 tau fibrils at 14 DIV. BSCs were photoconverted at 24 DIV (10 days after the addition of fibrils at 14 DIV) and then imaged at several time points from 24DIV (immediately post-photoconversion) until 45 DIV (21 days post-photoconversion). **c** Line graphs show amounts of photoconverted Dendra2 fluorescent intensity in seeded and non-seeded conditions that were quantified over time as a proportion of initial fluorescent intensity (*n* = 9, data are mean ± SEM, analyzed by two-way ANOVA with post hoc Sidak’s test, **p < 0.01, ****p < 0.0001). **d** Line graphs show amounts of Dendra2 fluorescent intensity in non-seeded and seeded conditions that were quantified over time as a proportion of initial fluorescent intensity (*n* = 9, data are mean ± SEM, *p < 0.05, **p < 0.01, two-way ANOVA with post hoc Sidak’s test)

Tau seeding and propagation have been a major focus of recent studies [23], but the precise dynamics of inclusions induced by seeding has not been established. After observing that exogenous K18 tau fibrils can induce P301L-tau-Dendra2 to form inclusions rapidly over a 10 day period, we aimed to compare the longevity of soluble non-seeded P301L-tau-Dendra2 tau to aggregated, seeded P301L-tau-Dendra2 using long-term optical pulse labeling experiments. A schematic diagram of the imaging timeline is shown in Fig. 7a. After 14 DIV, P301L-tau-Dendra2 BSCs were seeded with K18 tau fibrils, or untreated for a further 10 DIV, and at this point BSCs underwent photoconversion. The red fluorescent photoconverted Dendra2-tagged tau pool and newly synthesized green fluorescent Dendra2-tagged tau were tracked using long-term live imaging (Fig. 7b). Population level quantification to assess protein turnover (Fig. 7c) identified levels of photoconverted P301L-tau-Dendra2 in the presence of K18 tau fibrils were significantly higher than non-seeded P301L-tau-Dendra2 BSCs throughout the live imaging study from 1 to 21 days post photoconversion. Estimated half-lives of the photoconverted Dendra2-tagged P301L tau reveal P301L tau without seeds had a half-life of 2.72 days; 95% CI [2.34, 3.17], *r*^2^ = 0.97, whilst seeded P301L tau showed a half-life of 8.16 days; 95% CI [5.51, 14.01], *r*^2^ = 0.88. These findings highlight that seeded P301L-tau-Dendra2 has a significantly longer half-life than non-seeded P301L-tau-Dendra2. We also confirmed that the half-life of P301L-Dendra2 alone or in the presence of K18 tau fibrils does not significantly depend on initial fluorescent intensity or expression levels (Supp. Figure 3), as per previous reports [1]. Of note, the longevity and half-life of tau inclusions formed through the seeding of P301L-tau-Dendra2 was similar to that of inclusions formed through intrinsic P301L/S320F-tau-Dendra2 aggregation (Fig. 2c), suggesting inclusions comprised of aggregates formed either intrinsically or extrinsically show a similar turnover.

To estimate any effects of seeding on tau production, newly synthesized P301L-tau-Dendra2 was quantified (Fig. 7d) and found to accumulate at similar rates in the presence and absence of seeds. P301L-tau-Dendra2 seeded BSCs accumulated significantly higher newly synthesized tau 14 DIV after the initial photoconversion with non-seeded P301L-tau-Dendra2 showing a more rapid turnover of newly synthesized tau over these long-term imaging periods [similar to WT-tau-Dendra2 and Dendra2 alone (Fig. 2)].

These data suggest that seeding of P301L tau induces tau inclusions that show turnover at a slower rate than unseeded P301L tau, but the addition of K18 tau fibrils does not affect overall tau production.

**Fig. 8:**
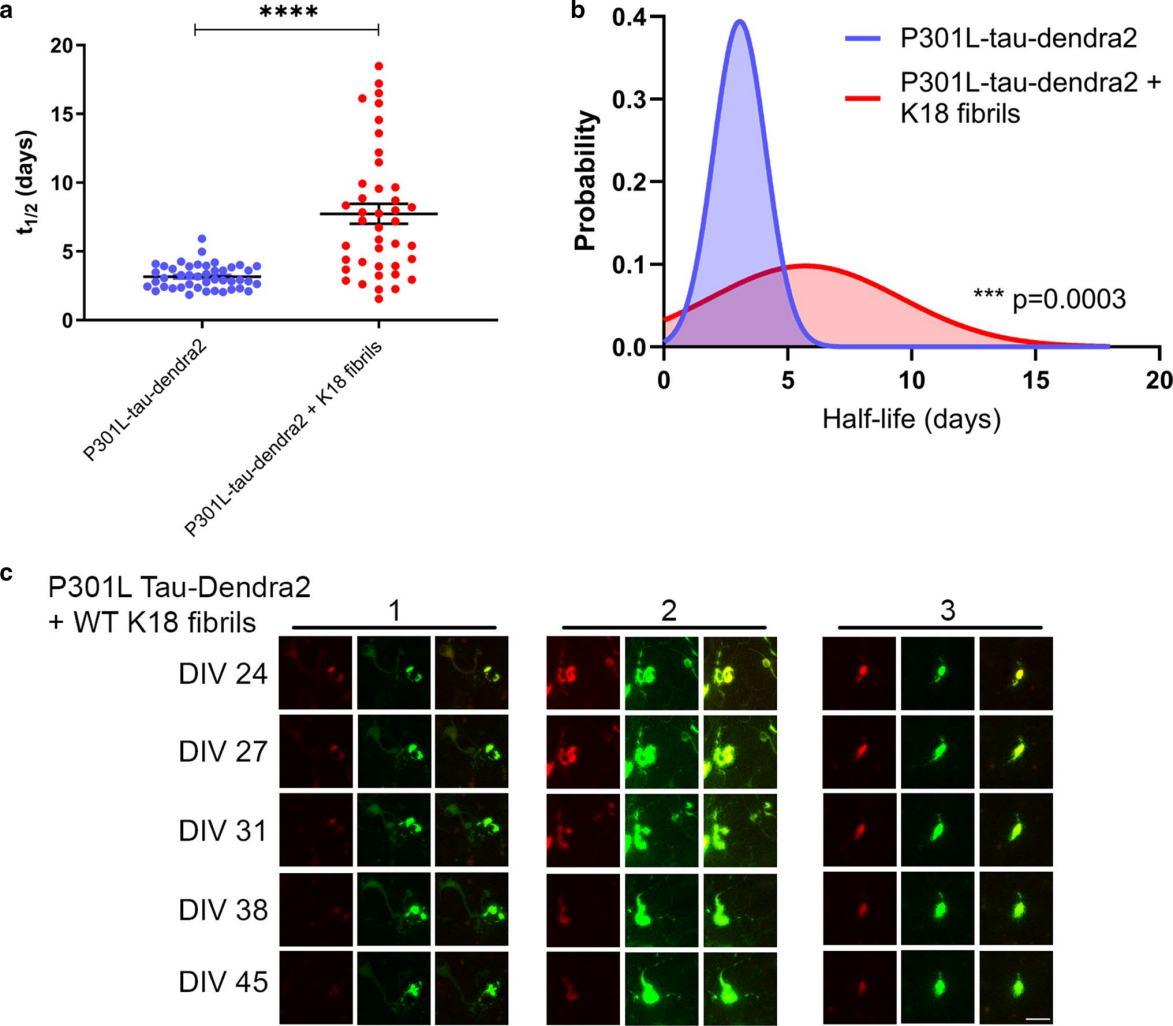
Clearance and decline in seeded P301L-tau-Dendra2 inclusions is attributed to inclusion turnover. **a** Scatter plot of half-life estimates for individual living cells in BSCs expressing P301L-Dendra2 ± K18 fibrils (*n* = 41–44; ****p < 0.0001, unpaired *T* test). **b** Probability density plot of half-life estimates for individual living cells expressing P301L-Dendra2 ± K18 fibrils (*n* = 41–44; *p = 0.0003, two-sided Kolmogorov–Smirnov test). Values were pooled from 5 BSCs per condition. **c** Representative images of three examples of P301L-Dendra2 + K18 fibril seeded cells which show turnover of photoconverted (red) tau in inclusions while newly synthesized (green) fluorescence demonstrates the cell is alive. Merge showing exchange of red and green P301L tau within individual cells after seeding is also shown. Scale bar = 25 µm

We then examined the fate of inclusions under P301L-tau-Dendra2 seeded conditions, where population analysis suggests some clearance of seeded tau inclusions, at an individual cellular level. For this single cell analysis, a sample of individual cells alive and emitting green fluorescence at 45 DIV at the end of the imaging period to omit any bias from cells that had died. This analysis of living individual cells identified the mean half-life of P301L-tau-Dendra2 at 3.161 days; 95% CI [2.90, 3.43], compared to P301L-tau-Dendra2 + K18 fibrils at 7.73 days; 95% CI [6.26, 9.20] (Fig. 8a). We also performed a probability density analysis, confirming the seeded P301L tau shows a significantly longer half-life (Fig. 8b). Representative images of individual tau inclusions in cells turning over in seeded P301L-tau-Dendra2 BSCs are shown in Fig. 8c. As observed in P301L/S320F-tau-Dendra2 BSCs, the red photoconverted tau, can be seen to decrease over time and be replaced by green fluorescence whilst the overall size of the fluorescent ‘inclusion’ appears to remain consistent or slightly increased in the seeded P301L-tau-Dendra2 BSCs.

### Extended time in culture increases the half-life of seeded P301L-tau-Dendra2 inclusions

**Fig. 9:**
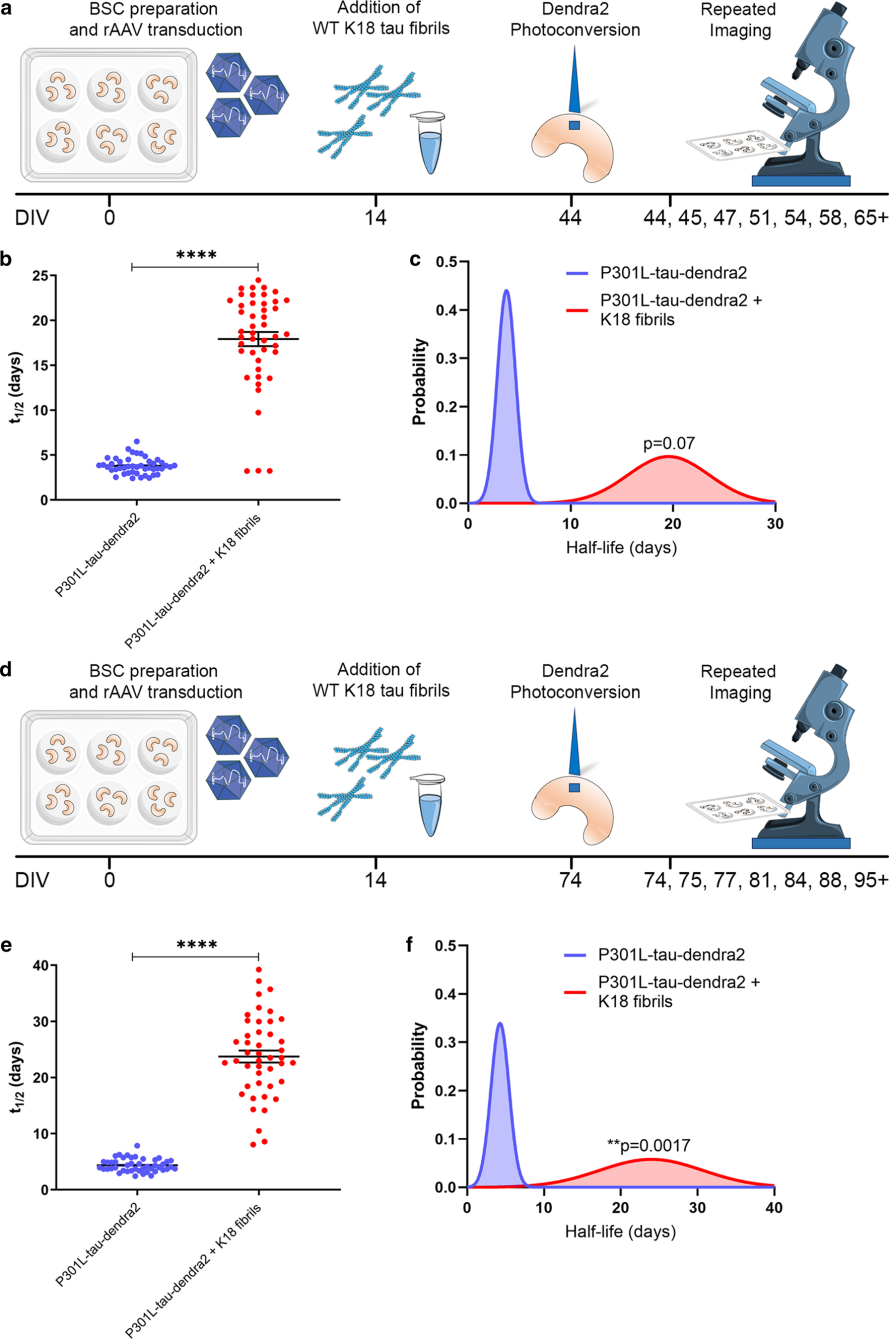
Seeded tau inclusions continue to turnover after extended periods in culture but at a slower rate. **a** A schematic diagram shows the timeline of the long-term optical pulse labeling experiments using photoconversion of P301L-tau-Dendra2 with and without K18 tau fibrils photoconverted at ~ 1 month after seeding. **b** Scatter plot of half-life estimates for individual living cells in BSCs expressing P301L-Dendra2 ± K18 fibrils (*n* = 45; ****p < 0.0001, unpaired *T* test). **c** Probability density plot of half-life estimates for individual living cells expressing P301L-Dendra2 ± K18 fibrils (*n* = 45; *p* = 0.07, two-sided Kolmogorov–Smirnov test). Values were pooled from 5 BSCs per condition. **c** A schematic diagram shows the timeline of the long-term optical pulse labeling experiments using photoconversion of P301L-tau-Dendra2 with and without K18 tau fibrils photoconverted at ~ 2 months after seeding. **d** Scatter plot of half-life estimates for individual living cells in BSCs expressing P301L-Dendra2 ± K18 fibrils (*n* = 45; ****p < 0.0001, unpaired *T* test). **e** Probability density plot of half-life estimates for individual living cells expressing P301L-Dendra2 ± K18 fibrils (*n* = 45; **p = 0.0017, two-sided Kolmogorov–Smirnov test). Values were pooled from 5 BSCs per condition

After identifying that tau inclusions in P301L-tau-Dendra2 BSCs formed 10 DIV after the addition of K18 tau fibrils turned over with a half-life of ~ 1 week, we sought to determine how ageing or longer durations in culture may affect the half-life and turnover of these inclusions. A schematic diagram of the long-term imaging timeline after longer exposure to inclusions and K18 tau fibrils is shown in Fig. 9a. We tracked the red fluorescent photoconverted Dendra2-tagged tau pool using long-term live imaging after photoconverting at ~ 1 month after the addition of K18 tau fibrils.

We determined in individual living cells, the rate of tau turnover and replacement in BSCs that had been aged for ~ 1 month after the addition of K18 tau fibrils prior to photoconversion. This analysis identified the mean half-life of P301L-tau-Dendra2 at 3.80 days; 95% CI [3.53, 4.07], compared to P301L-tau-Dendra2 + K18 fibrils at 17.92 days; 95% CI [16.32, 19.52] (Fig. 9b) ~ 1 month after the addition of fibrils. We also performed a probability density analysis between the two conditions (Fig. 9c).

We then extended the time in culture after fibril addition prior to photoconversion and long-term imaging to ~ 2 months—a schematic diagram of this timeline is shown in Fig. 9d. This analysis identified the mean half-life of P301L-tau-Dendra2 at 4.36 days; 95% CI [4.02, 4.71], compared to P301L-tau-Dendra2 + K18 fibrils at 23.73 days; 95% CI [21.57, 25.90] (Fig. 9e) ~ 2 months after the addition of fibrils. Probability density analysis confirmed the seeded P301L-tau-Dendra2 shows a significantly longer half-life (Fig. 9f).

Taken together, these findings indicate that extended culture periods or exposure to a tau inclusion after seeding increases the half-life of the inclusion turnover 1 month post seeding to approximately 2 weeks, and 2 months post seeding to approximately 3 weeks. This further underscores the similarities between intrinsic and seeded tau inclusions and the slowing down of tau turnover over time in these models.

## Discussion

Using long-term optical pulse labeling studies in BSCs we find that pro-aggregant P301L/S320F-tau-Dendra2 forms neuronal tau inclusions rapidly (12–96 h) and that these inclusions once formed show appreciable daily turnover with an overall half-life of ~ 7 days. In contrast, WT-tau-Dendra2 does not form inclusions and turns over with a half-life of ~ 3 days. This turnover of P301L/S320F-tau-Dendra2 is observed as both decreases in red photoconverted species and replacement within the inclusion by more recently synthesized green non-photoconverted tau. Rapid formation of inclusions is also observed in a newly developed ‘seeded’ P301L-tau BSC model, which only forms inclusions in the days following the addition of exogenous K18 tau fibrils, but also turns over with a half-life of ~ 7 days. This rapid formation of tau inclusions in BSCs is reminiscent of the formation of β-amyloid plaques observed in vivo [42] and on a timescale of tau inclusion formation in vivo in tau transgenic mice [18]. We also find that the half-life of tau inclusions increases and tau protein turnover slows down upon aging for longer durations in culture.

The evidence that tau inclusions can turnover is somewhat surprising, given that tau inclusions appear to persist within neurons for many months in animal models and years (or even decades) in humans [3]. However, the size of tau inclusions must clearly be limited by processes occurring within the cell, as inclusions do not invariably fill spaces much larger than the soma of the cells in which they form. Thus, neurons appear to adapt to inclusion formation in a way that enables them to live and function within relatively normal parameters. Indeed, others have shown that cells with tau inclusions can exist amongst functional neuronal circuits and the presence of tau aggregates does not markedly affect neuronal signaling function, at least within the time period studied [36, 44, 47]. Our data suggest that, the cells limit inclusion growth by dynamically turning the inclusion over and replacing with newly synthesized tau. This is best observed in cells that initially bear large amounts of photoconverted P301L/S320F-tau-Dendra2 in the soma or seeded P301L-tau-Dendra2 tau with a half-life of ~ 7 days which is then slowly replaced over days to weeks by non-photoconverted tau, yet the size of the overall inclusion remains approximately the same. Upon aging in culture, tau aggregate turnover continues to occur but at a slower rate. Previous in vivo and in vitro studies have shown free tau has a half-life of hours in stably and transiently transfected cell cultures, days in iPSC-derived neurons, and weeks in tau transgenic mice and in human CSF [16, 49, 54, 55]. Our current studies show that tau in both ‘intrinsic’ and ‘seeded’ inclusions can also be turned over and replaced.

Many unanswered questions that these studies raise can be addressed using paradigms developed here or other complementary methods in the future. Newly formed inclusions turnover relatively quickly, but as the BSCs are aged the rate of turnover of tau decreases. Whether this half-life extension reflects a change in the tau aggregate, a change in the cellular response to the tau inclusion, or some combination of the two will need to be explored. Even in the older cultures, there is a wide distribution of inclusion turnover rates for individual cells. Could understanding this variability of turnover provide insight into selective vulnerability to tau inclusion pathology? Further, we document a progressive increase in tau inclusion half-life as the cultures age. Does this half-life prolongation continue in even older cultures? Or does it plateau? Clearly, there will be some limitations of our current model systems, where we would likely only be able to look at inclusions that are up to 1 year old [10, 11, 17]. Answering these questions will be important as the answers will help to define the window where future therapeutics designed to enhance turnover of inclusions might be most effective [25].

This data and a previous study that hint that insoluble tau species in vivo in tau transgenic also turnover [55] have potentially important therapeutic implications. Most tau targeting therapeutics developed to date (e.g., antibodies, antisense oligonucleotides, small molecules that block aggregation) are designed to prevent tau inclusion formation. The demonstration that tau inclusions can turnover, indicate that it may be possible to clear inclusions, at least those that are relatively newly formed. Future studies that can provide insights into the mechanism by which inclusions are turned over may well provide new therapeutic strategies and targets to treat tauopathies.

Importantly, optical pulse labeling enables an accurate estimate of half-life of tau in inclusions in individual living cells avoiding population-based assays such as metabolic pulse-chase which are susceptible to potential confounds such as cell death [1]. Though cells with tau inclusions can die [3, 48], the rate of cell death is not sufficient to account for the overall turnover of photoconverted pro-aggregant tau as evidenced by single-cell analysis of individual living cells with inclusions.

For future tau therapies, strategies to restrict replacement of newly produced tau to the inclusion may also be evaluated. Of course, as we still do not understand the relationship between inclusion formation and functional impact on neuronal physiology and cell death, we must also ensure that our studies not only focus on altering tau inclusion pathology but show beneficial effects on cellular function and block degeneration [3, 28, 36]. Nevertheless, this methodology provides a powerful tool which can be adopted to monitor how therapies may modulate tau turnover and whether this is linked to functional benefit.

In summary, this work exemplifies a simple and efficient platform which enables the tracking of tau inclusion formation and turnover through long-term optical pulse labeling studies in different models of tau pathology, revealing neurons can turn over and replace tau within inclusions. Furthermore, our work directly suggests that tau in individual inclusions may be cleared even after prolonged durations bearing an inclusion, giving hope that therapies which modulate tau production or clearance may be able to stimulate this turnover of tau in inclusions and be beneficial in the treatment of tauopathies. Overall, by using this approach we can begin to delineate mechanisms mediating this inclusion turnover which may lead to new therapies for the treatment of tauopathies.

## Supplementary Information

Below is the link to the electronic supplementary material.

Supplementary Figure 1. Half-life of Dendra2 does not significantly depend on initial fluorescent intensity expression levels. BSCs expressing (a) Dendra2, (b) WT-tau-Dendra2 or (c) P301L/S320F-tau-Dendra2 were photoconverted at 10 DIV and initial fluorescence intensity at a population level was measured. These values were then plotted against the half-life of these same cell populations and linear regression analysis performed. The correlation coefficients (*r*^2^) and P values are shown and indicate that the half-life of the fluorescent cell populations is independent of their initial fluorescent intensity levels. (n=12) (TIF 273 KB)

Supplementary Figure 2. P301L/S320F-tau-Dendra2 inclusions form rapidly in neurons. BSCs were prepared and transduced with rAAVs to express P301L/S320F-tau-Dendra2 exclusively in neurons using a neuronal promoter (MAP-2) on 0 days in vitro (DIV) and then maintained in culture until 28 DIV to confirm inclusions form in neurons in BSCs. (a) Transduced BSCs were fixed and stained with Thiazin Red to identify any β-sheet structures in neurons expressing P301L/S320F-Dendra2. Scale bar = 50 µm. (n=3). (b) Representative images of MAP-2-P301L/S320F-tau-Dendra2 emitted green fluorescence imaged live from 21 DIV highlights neurons develop inclusions in the soma over a period of hours (fast), or over a period of several days (slow) and then continue to exist for at least 7 days once formed. Scale bars = 50 µm (TIF 1407 KB)

Supplementary Figure 3. Half-life of P301L-tau-Dendra2 does not significantly depend on initial fluorescent intensity expression levels. BSCs expressing P301L-tau-Dendra2 (a) without fibrils and (b) seeded with K18 fibrils were photoconverted at 24 DIV (10 DIV after seeding) and initial fluorescence intensity at a population level was measured. These values were then plotted against the half-life of these same cell populations and linear regression analysis performed. The correlation coefficients (*r*^2^) and P values are shown and indicate that the half-life of the fluorescent cell populations is independent of their initial fluorescent intensity levels. (n=9) (TIF 229 KB)

## Abbreviations

AD: Alzheimer’s disease
BME: Basal medium eagle
BSA: Bovine serum albumin
BSC: Brain slice culture
CNS: Central nervous system
DIV: Days in vitro
DMSO: Dimethylsulfoxide
FTDP-17: Frontotemporal dementia with Parkinsonism linked to chromosome 17
HBSS: Hank’s balanced salt solution
hCBA: Hybrid cytomegalovirus enhancer/chicken β-actin (hCBA) promoter
MAPT: Microtubule-associated protein tau
MTBD: Microtubule binding domain
NFT: Neurofibrillary tangle
rAAV: Recombinant adeno-associated virus
WT: Wild-type
