# Supplementary figures and images for: Photodynamic studies reveal rapid formation and appreciable turnover of tau inclusions
Source: Acta Neuropathol. 2021 Jan 26;141(3):359–81. doi: 10.1007/s00401-021-02264-9 (PMC7882582; doi:10.1007/s00401-021-02264-9)

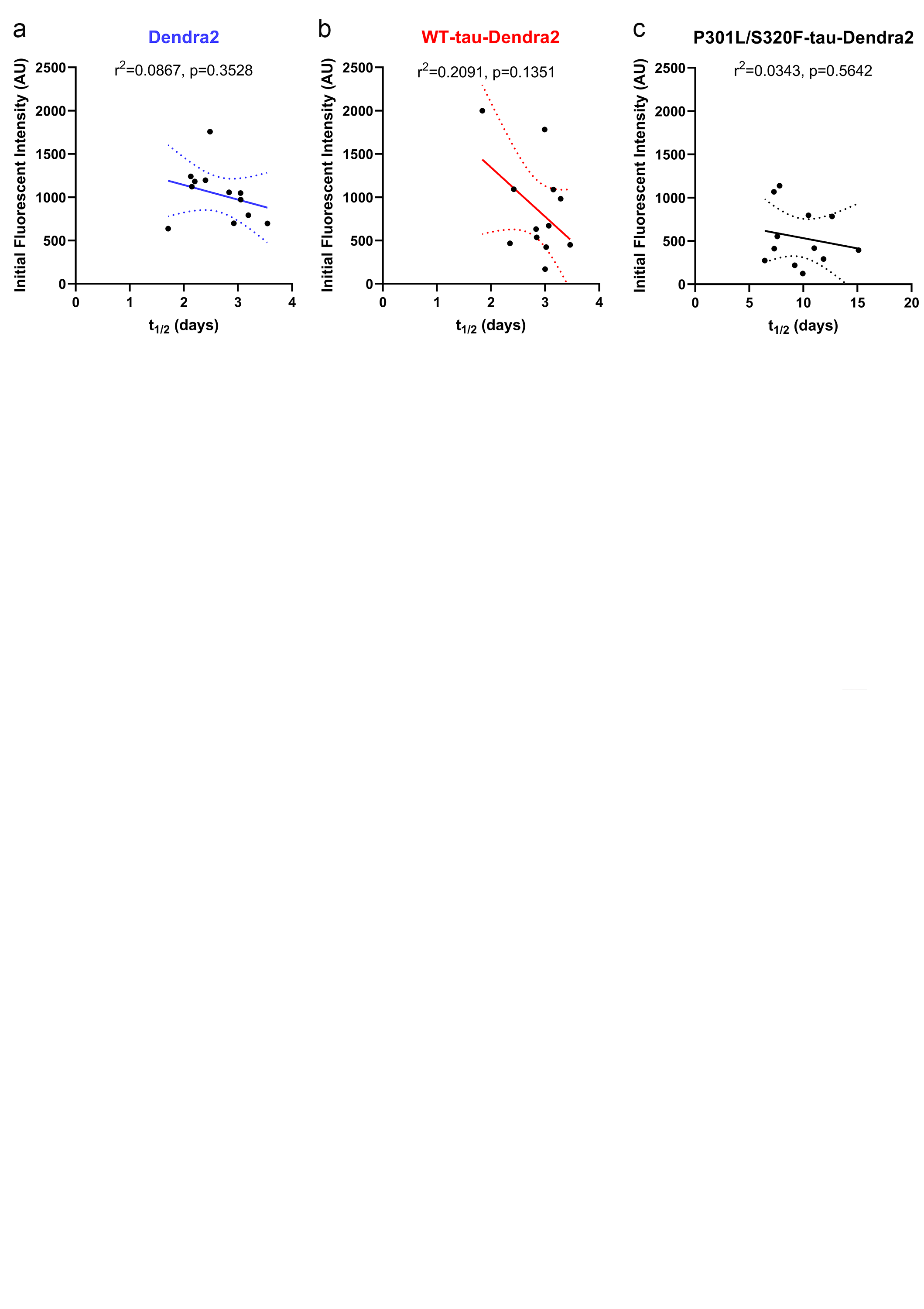

Supplement: Supplementary file 1 — Supplementary Figure 1. Half-life of Dendra2 does not significantly depend on initial fluorescent intensity expression levels. BSCs expressing (a) Dendra2, (b) WT-tau-Dendra2 or (c) P301L/S320F-tau-Dendra2 were photoconverted at 10 DIV and initial fluorescence intensity at a population level was measured. These values were then plotted against the half-life of these same cell populations and linear regression analysis performed. The correlation coefficients (r2) and P values are shown and indicate that the half-life of the fluorescent cell populations is independent of their initial fluorescent intensity levels. (n=12) (TIF 273 KB) [file 401_2021_2264_MOESM1_ESM.tif]

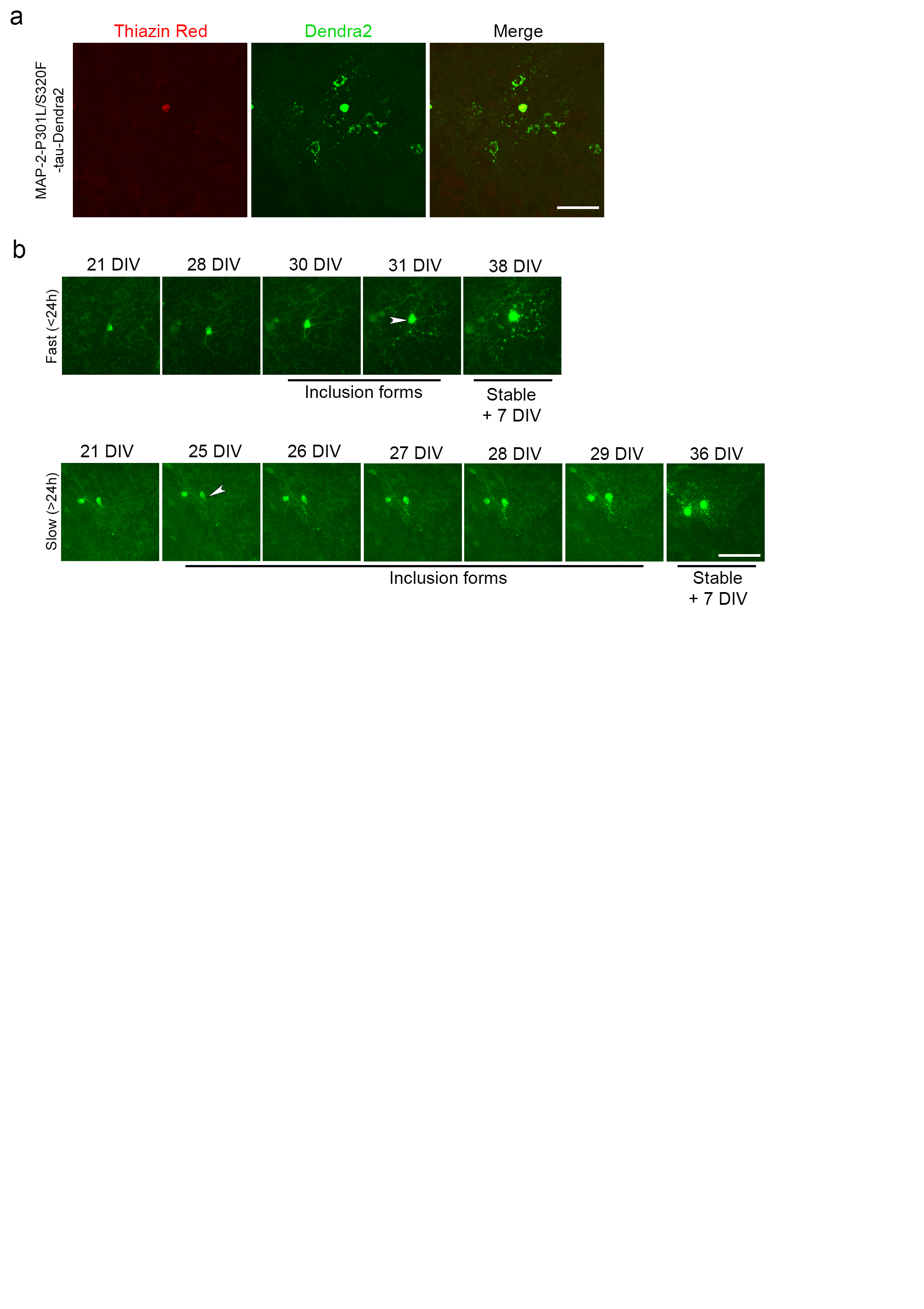

Supplement: Supplementary file 2 — Supplementary Figure 2. P301L/S320F-tau-Dendra2 inclusions form rapidly in neurons. BSCs were prepared and transduced with rAAVs to express P301L/S320F-tau-Dendra2 exclusively in neurons using a neuronal promoter (MAP-2) on 0 days in vitro (DIV) and then maintained in culture until 28 DIV to confirm inclusions form in neurons in BSCs. (a) Transduced BSCs were fixed and stained with Thiazin Red to identify any β-sheet structures in neurons expressing P301L/S320F-Dendra2. Scale bar = 50 µm. (n=3). (b) Representative images of MAP-2-P301L/S320F-tau-Dendra2 emitted green fluorescence imaged live from 21 DIV highlights neurons develop inclusions in the soma over a period of hours (fast), or over a period of several days (slow) and then continue to exist for at least 7 days once formed. Scale bars = 50 µm (TIF 1407 KB) [file 401_2021_2264_MOESM2_ESM.tif]

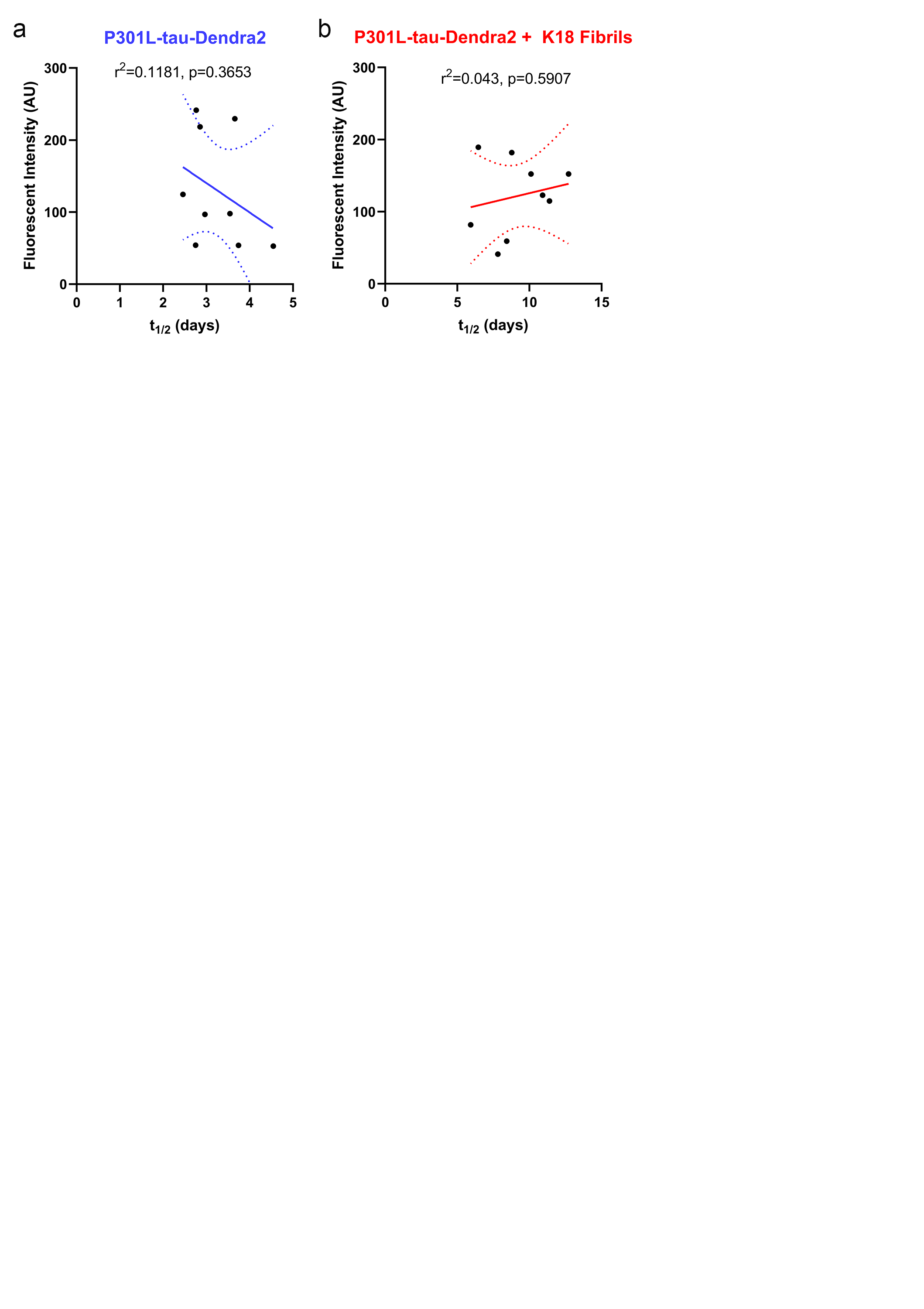

Supplement: Supplementary file 3 — Supplementary Figure 3. Half-life of P301L-tau-Dendra2 does not significantly depend on initial fluorescent intensity expression levels. BSCs expressing P301L-tau-Dendra2 (a) without fibrils and (b) seeded with K18 fibrils were photoconverted at 24 DIV (10 DIV after seeding) and initial fluorescence intensity at a population level was measured. These values were then plotted against the half-life of these same cell populations and linear regression analysis performed. The correlation coefficients (r2) and P values are shown and indicate that the half-life of the fluorescent cell populations is independent of their initial fluorescent intensity levels. (n=9) (TIF 229 KB) [file 401_2021_2264_MOESM3_ESM.tif]
